# Soft ionization mechanisms in flexible µ-tube plasma—from FµTP to closed µ-tube plasma

**DOI:** 10.1007/s00216-024-05420-8

**Published:** 2024-07-03

**Authors:** Luisa Speicher, Hao Song, Norman Ahlmann, Daniel Foest, Simon Höving, Sebastian Brandt, Guanghui Niu, Joachim Franzke, Caiyan Tian

**Affiliations:** 1 Leibniz-Institute for Analytical Sciences – ISAS – eV., Bunsen-Kirchhoff-Straße 11, 44139 Dortmund, Germany; 2 Laboratory of Inorganic Chemistry, Department of Chemistry and Applied Biosciences, ETH Zurich, Vladimir-Prelog-Weg 1, 8093 Zurich, Switzerland

**Keywords:** New soft ionization source, Flexible µ-tube plasma (FµTP), Long µ-tube plasma (LµTP), Closed µ-tube plasma (CµTP)

## Abstract

In order to find an explanation for the mechanism in a plasma operated with an alternating voltage, or rather a square wave voltage, such a plasma was investigated. It was found that Penning ionization, charge transfer, and photoionization played a minor or even no role in the soft ionization mechanism of a FµTP. If the collision of plasma gases with air does not contribute to soft ionization, it should also be possible to use a separated plasma for soft ionization. Preliminary investigations show that it is possible to ignite a diagnosis gas with a plasma gas even when there is a barrier such as glass between those gases. A temporally and locally limited potential must be produced at the outer surface to achieve this. This potential should be sufficient to ionize the environment softly and to be able to use this so-called closed µ-tube plasma as a new ionization source.

**Supplementary Information:**

The online version contains supplementary material available at 10.1007/s00216-024-05420-8.

## Introduction

Plasma-based ionization sources have been widely used for analysis of various samples performed in front of the mass spectrometer inlet. In most cases, high-purity helium is used as a plasma gas such as DBDI [1], LTP [2], DART [3], and FAPA [4]. The ionization mechanism for He-based sources seems to be understood fully, where N_2_^+^ ions are produced by Penning ionization with He^M^ or charge transfer from He^+^ or He_2_^+^ [5]. These N_2_^+^ ions generate (H_2_O)_n_H^+^ by a series of reactions. Or the H_2_O^+^ ions are produced directly by Penning ionization, and eventually the samples are protonated.

Argon is becoming a more economical option as a discharge gas instead of He for the analysis of sample with a mass spectrometer. Ar plasmas can compete with He plasmas and sometimes the ionization efficiency is higher than that of He plasmas [6, 7]. The mechanism related to Ar plasmas has not been clarified so far and further studies are needed, as no N_2_^+^ nor H_2_O^+^ can be generated by Penning ionization as happening in He plasmas.

Recently, not only the ionization efficiency of He, Ar, Kr, and Xe plasmas as well as the photoionization lamps has been evaluated and compared, but also the discharge and propagation mechanism of plasmas have been investigated by means of optical emission spectroscopy to figure out the soft ionization mechanism [8]. These plasmas were generated in a flexible µ-tube plasma (FµTP) ionization source without changing the dimensions of the housing. According to the results obtained by mass spectrometry, it was demonstrated that Penning ionization and charge transfer between reactive species of plasma and ambient air/analytes as well as photoionization are not dominant soft ionization process.

In the case of He-FµTP, N_2_^+^ ions are the dominant ions promoting the excitation and ionization propagation forward, while in other plasmas, noble gas ions play a role in the propagation through the discharge capillary. These ions stop at the end of the capillary, while the excitation wave propagates further beyond the capillary end to the ambient air. There must be an ion cloud, which is following the propagation of excitation wave or in other words, there must be an ion cloud which attracts and accelerates electrons forming excited states in front of the ion cloud. Otherwise, the excitation wave will stop. Introducing a stream of diagnosis gas to the position where the analytes are usually placed, the diagnosis gas with higher excited energy is ignited by Ar-, Kr- and Xe-FµTPs with lower excited energies, even ionization energies.

Based on these findings, we proposed an alternative mechanism that a transient potential created by ions is mainly responsible for the soft ionization, which is not dependent on the type of the discharge gas, but the number of the ions at the end of the capillary. The expression temporally limited potential reminds of a process known from dielectric barrier discharges, where a fast-changing potential conditions the process.

In this work, to dispel any remaining doubts that neither Penning ionization, charge transfer, nor photoionization is responsible for the soft ionization, we present further experimental arrangements, where a glass wall either that of the diagnosis capillary or that of the discharge capillary separates the discharge gas from the diagnosis gas. In the following preliminary investigation, this will be presented.

### Preliminary investigation: plasma development beyond a glass wall

It has been shown in our previous study [8] that the Xe-FµTP with the highest energy state of 12.2 eV (Xe^+^) is able to ignite He with an energy of 22.7 eV when the two gases come from two different tubes and have a crossing point. It was assumed that these positive charges are formed behind the orifice of the plasma capillary to establish (a time-dependent) transient potential and therewith an electric field. This field should be high enough to attract and accelerate electrons which then collide with for example water molecules generating protons like H_2_O^+^ or be high enough to polarize water molecules and to extract electrons from them leaving H_2_O ions H_2_O^+^. If one or both mechanisms are true, and there will be a real transient potential (several 100 ns) outside the FµTP capillary, it should be able to ionize other gases even when there is a dielectric barrier between it.

**Fig. 1:**
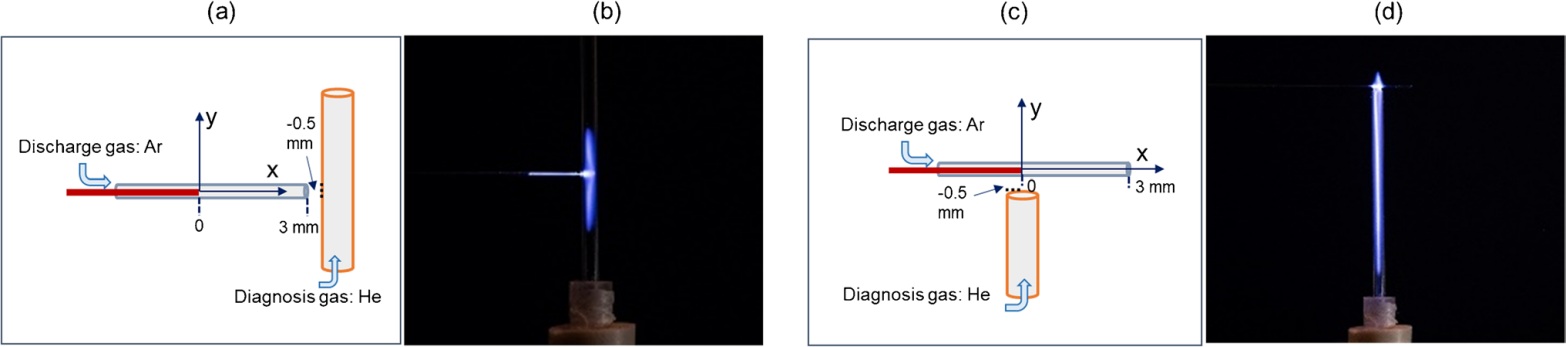
Experimental arrangements and plasma development beyond a glass wall. The glass wall of diagnosis tube is located between two gases (**a**) and the corresponding photo of plasma emission (**b**). The glass wall of discharge capillary is located between two gases (**c**) and the corresponding photo of plasma emission (**d**). He is used as diagnosis gas triggered by an Ar-FμTP

Two experiments were carried out to prove that. In both cases, a glass is positioned as a dielectric barrier between the volume to be ionized and the discharge plasma. The volume to be ionized is inside a so-called diagnosis tube. In one case, the diagnosis tube is shifted so far along the y axis that the glass wall of the diagnosis tube is between the discharge gas flow and the diagnosis gas flow. In the other case, the diagnosis tube is shifted along the x axis so that the glass wall of the discharge capillary is located between the discharge gas and the diagnosis gas. In both cases, the used gases have no contact to each other, they can be handled as two individual gas flows without any mixture of them. The one in the plasma capillary is called plasma gas and the other one diagnosis gas. Figure 1a shows the experimental arrangement for the first case, using an exciting Ar plasma and the emission running vertically in the diagnosis tube with a He flow rate of 25 mL min^−1^. In Fig. 1b, the corresponding photo is displayed. It can be seen that even with a glass wall between the Ar-FµTP and the diagnosis gas He emission is visible inside the diagnosis tube. Figure 1c shows the experimental arrangement of the second case with a photograph (Fig. 1d) of the Ar-FµTP where the He emission runs perpendicular to the axis of the discharge capillary in the diagnosis tube with a He flow rate of 500 mL min^−1^.

Therefore, it is not necessary to have a contact between plasma gas and diagnosis gas. In this manner, the Penning ionization and the charge transfer between the discharge plasma and diagnosis gas for the excitation of diagnosis gas can be completely excluded. A potential is formed outside the capillary which can be explained by H_2_O^+^ being produced in the vicinity of the orifice for the first case. The second case shows that a transient potential is also formed around the whole plasma tube. It can be assumed that the ion wave polarizes the glass wall of the capillary and the potential change at the outer wall is high enough to induce an electric field to generate H_2_O^+^.

Based on this, a new ionization technique is proposed. The FµTP is modified to use it without the plasma jet and further a closed plasma is developed. It is working without any noble gas flow, just a noble gas inside a sealed glass piece and an integrated electrode is required. Different noble gases in the FµTP will be compared by using a mass spectrometer coupled to the modified long µ-tube plasma (LµTP) and a sealed closed µ-tube plasma (CµTP).

## Material and methods

### Ionization source and mass spectrometer

**Fig. 2:**
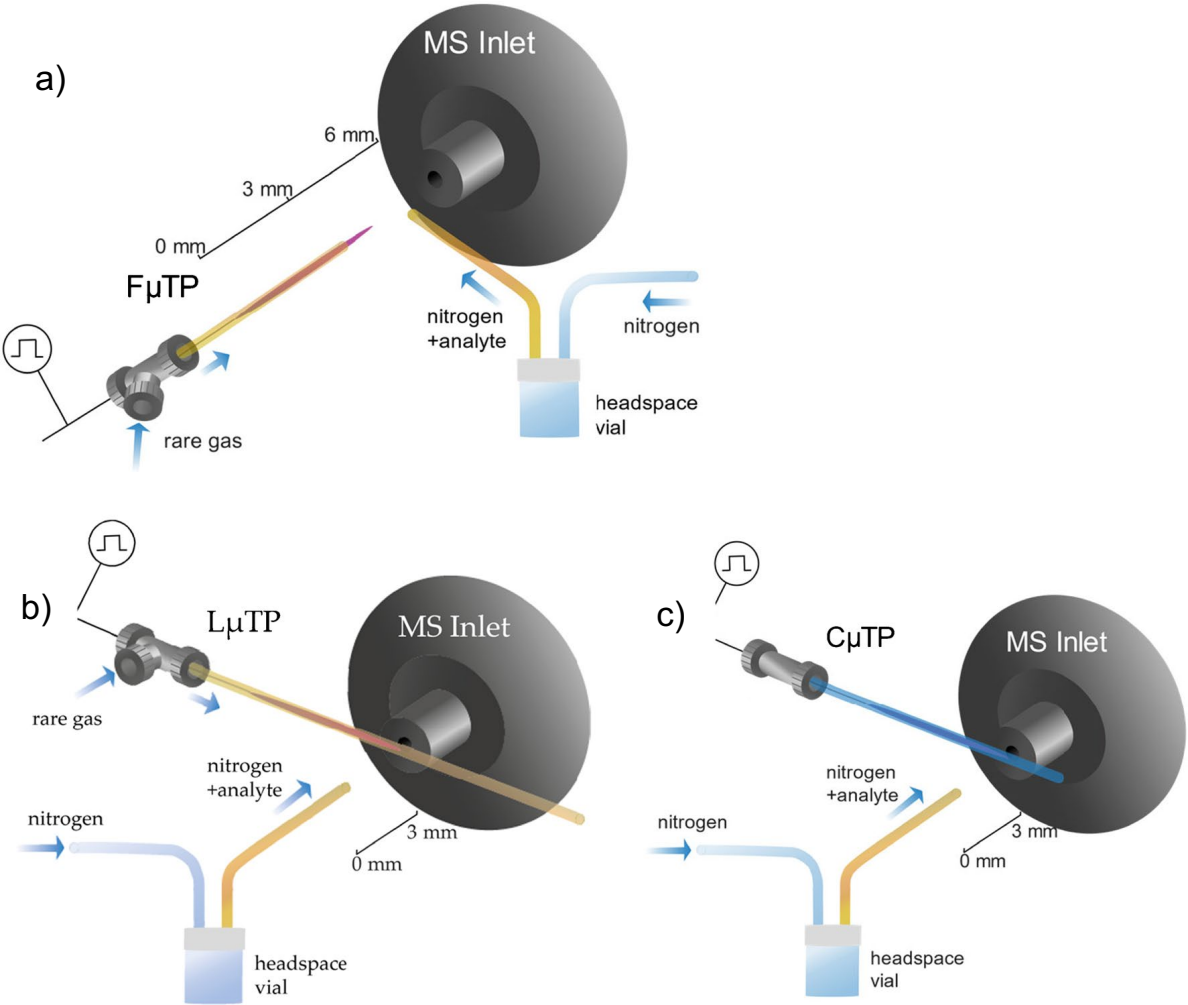
Experimental arrangement of **a** FµTP, **b** LµTP, and **c** CµTP

The polyimide-coated fused silica capillary of a FµTP has an inner diameter of 250 µm and an outer diameter of 350 µm. The electrode with a diameter of 100 µm lies inside the capillary and the distance between the electrode tip and the end of the capillary is 3 mm (Fig. 2a).

To initially mimic a closed plasma, the distance between the electrode tip and the end of the capillary was increased to about 150 to 200 mm so that the plasma is located inside the capillary (Fig. 2b). The polyimide layer was removed. To distinguish the plasmas to be compared, this plasma is called the long µ-tube plasma (LµTP). The LµTP was used with a gas flow of 75 mL min^−1^ with the discharge gases He 5.0 (purity 99.999%), Ar 5.0 (purity 99.999%), and Kr 5.0 (purity 99.999%). The tungsten wire inside the capillary is used as a high voltage electrode driven by a square wave voltage of 2.5, 3.0, or 3.5 kV peak to peak with a frequency of 20 kHz. The capillary of the LµTP was placed with its axis perpendicular to the axis of a Finnigan LTQ mass spectrometer inlet and is in direct contact with it. The distance between the electrode tip and the point of contact of the capillary with the MS inlet is 10 mm as shown in Fig. 2b. The capillary was heated to 200 °C, while the voltage was set to 11 V and the tube lens to 35 V which resulted in a tune on the (H_2_O)_2_H^+^ peak with a mass of m/z 37. Another fused silica capillary applied as headspace capillary to supply analytes was aligned on the axis of the MS inlet with 5 mm to it. The headspace flow was 100 mL min^−1^ of N_2_ while 2,5-hexanedione (HexD) obtained from Sigma-Aldrich was used as an analyte. The reactant gas passed over the liquid analyte in a vial. After the vapor pressure balanced, the headspace gas was used as the analyte gas.

Due to the extension of the capillary, no plasma gas will enter the inlet of the MS and it can be proven that no collision of the plasma with the ambient air is necessary to perform soft ionization. This LµTP was used as a representative for a closed version of a plasma sealed from the ambient air, where the production is not trivial and can lead to impurities in the gas.

The newly developed closed versions of the µTP were also placed in front of the LTQ (Fig. 2c). These plasmas were produced by using borosilicate capillaries with an outer diameter of 400 µm and an inner diameter of 300 µm. A tungsten wire is inserted on one side of the capillary. Under a gas flow using the same discharge gases He, Ar, and Kr as before, both capillary sides are closed by melting the glass capillary. This kind of plasma is called a closed µ-tube plasma (CµTP). It is currently still in a prototype phase, making it hard to get comparable CµTP regarding length and therewith volume. The used CµTP were placed with the same orientation in front of the MS as the LµTP, contacting it, with about 5 mm between touching place and the end of the wire which can be seen in Fig. 2c.

For comparing the ionization efficiencies of the two plasmas with that of a FµTP, the measurements were performed using the experimental arrangement shown in Fig. 2c, where the axis of the FµTP capillary overlaps with that of the LTQ inlet. A headspace capillary to apply the samples is aligned on the axis of the MS in between the plasma and the inlet which have a distance of 3 mm.

### Data acquisition and processing

The measured data for each shown spectrum is an average of 1 min of full scan mode measured with the LTQ mass spectrometer. The generated raw files were extracted from Xcalibur Qual browser as a txt file and the visualised via Python.

## Results and discussion

### Reference measurement with the FµTP

**Fig. 3:**
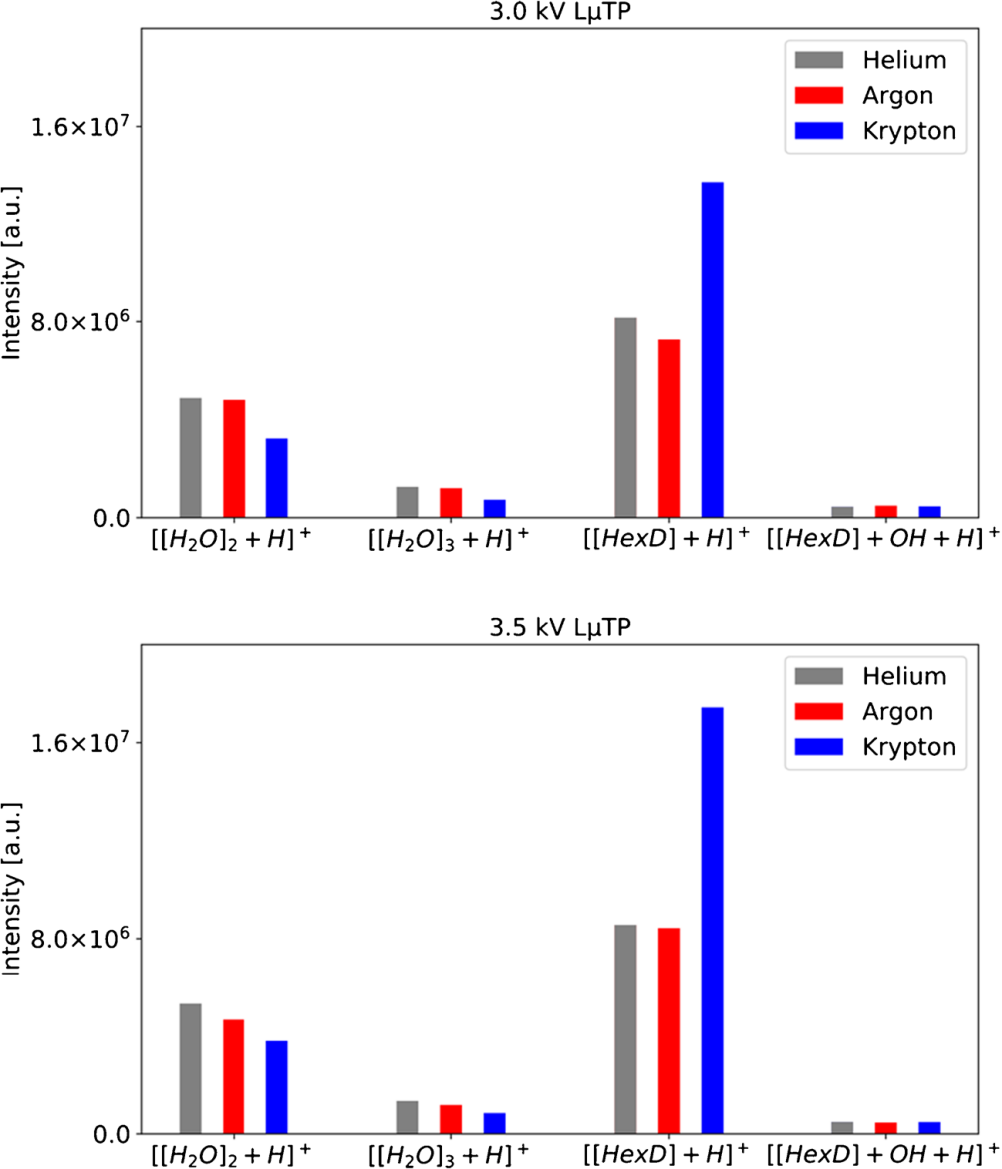
Peak intensities of [[H_2_O]_2_ + H]^+^, [[H_2_O]_3_ +]^+^, [HexD] + H]^+^, and [[HexD] + OH + H]^+^ obtained with He-, Ar-, and Kr-FµTP sources operated by 2.5 kV and 3.0 kV

For mass spectrometric characterization with respect to the ionization efficiency of the CµTPs, reference measurements were firstly performed with FµTPs of different noble gases and only one analyte (hexanedione (HexD)). The mass spectra are shown in Figure S1 and the corresponding peak intensities in Table S1; [H_2_O]_2_ H^+^ at m/z 37 and [H_2_O]_3_ H^+^ at m/z 55 are the blank signals representing the signals of protonated water when no analyte is induced. Together with the analyte HexD, the intensities of the peaks [HexD + H]^+^ at m/z 115 and [HexD + OH + H]^+^ at m/z 132 can be found in the named table. The measurements were performed with two different voltages of 2.5 and 3.0 kV. Since the intensities measured with the FµTPs operated with the noble gases He, Ar and Kr are in the same range, a mean value is given in Table S1. Only a slight increase in the ionization efficiency can be observed for He and Kr when the voltage is changed from 2.5 to 3.0 kV. In the case of argon, just the [HexD + H]^+^ increases, while the water peaks decrease. Table S1 is presented as a bar chart, shown in Fig. 3, with the noble gases used for plasma operation shown in different colors, it is apparent that the charts obtained with 2.5 and 3.0 kV hardly differ in their shape. The reason for this could be that an increase in voltage could only lead to higher potentials when the number of possible species such as H_2_O is high enough to form more corresponding ions like [H_2_O]_2_ H^+^ at m/z 37 and [H_2_O]_3_ H^+^ at m/z 55. If the number density of these species is not high enough, the signal might be saturated.

### Long µ-tube plasma source as physical representative for a closed plasma

Unlike the FµTPs where a short plasma jet is present in front of the MS inlet, and the axis of the FµTP capillary is aligned with the axis of the MS inlet, the plasma generated in LµTP is completely confined inside the capillary because of the extended length of the LµTP capillary. It is positioned perpendicular to the axis of the MS inlet. Thus, the end of the capillary is too far away from the inlet of the MS that collisions of excited or ionized species of the plasma with those of the ambient air can no longer play a role, meaning that Penning ionization or charge transfer between these reactive species of plasma and ambient air cannot take place. Therefore, this experimental arrangement is representative of a CµTP. The production of a CµTP is not trivial, because during a noble gas flow a capillary has to be melted off at two points. At one of the two points to be melted down, the introduced electrode must also be melted down. This process must be carried out without air penetrating into the CµTP.

When the capillary of LµTP is not in contact with the MS inlet, the ionization efficiency of LµTPs is poor, whereas when they are in contact, the ionization efficiency increases. This might be caused by the enhancement of the discharge process in the capillary, as the MS inlet acts as a second electrode. Therefore, the arrangement is comparable with a dielectric barrier discharge where two electrodes are involved [9, 10]. To investigate this, further emission spectroscopy measurements were made. A fast intensified charge coupled device (ICCD camera, Andor DH 720 18F-03) captured the temporally and spatially resolved spectra from a Kymera adaptive focus imaging spectrograph. An entrance slit of 200 µm and a 75-mm focal length lens were used. The LµTP was placed parallel to the entrance slit of the ICCD camera and was measured with and without a small copper metal sheet touching the outside of the plasma. The metal sheet was grounded and placed 5.5 cm away from the end of the electrode inside the fused silica capillary.

**Fig. 4:**
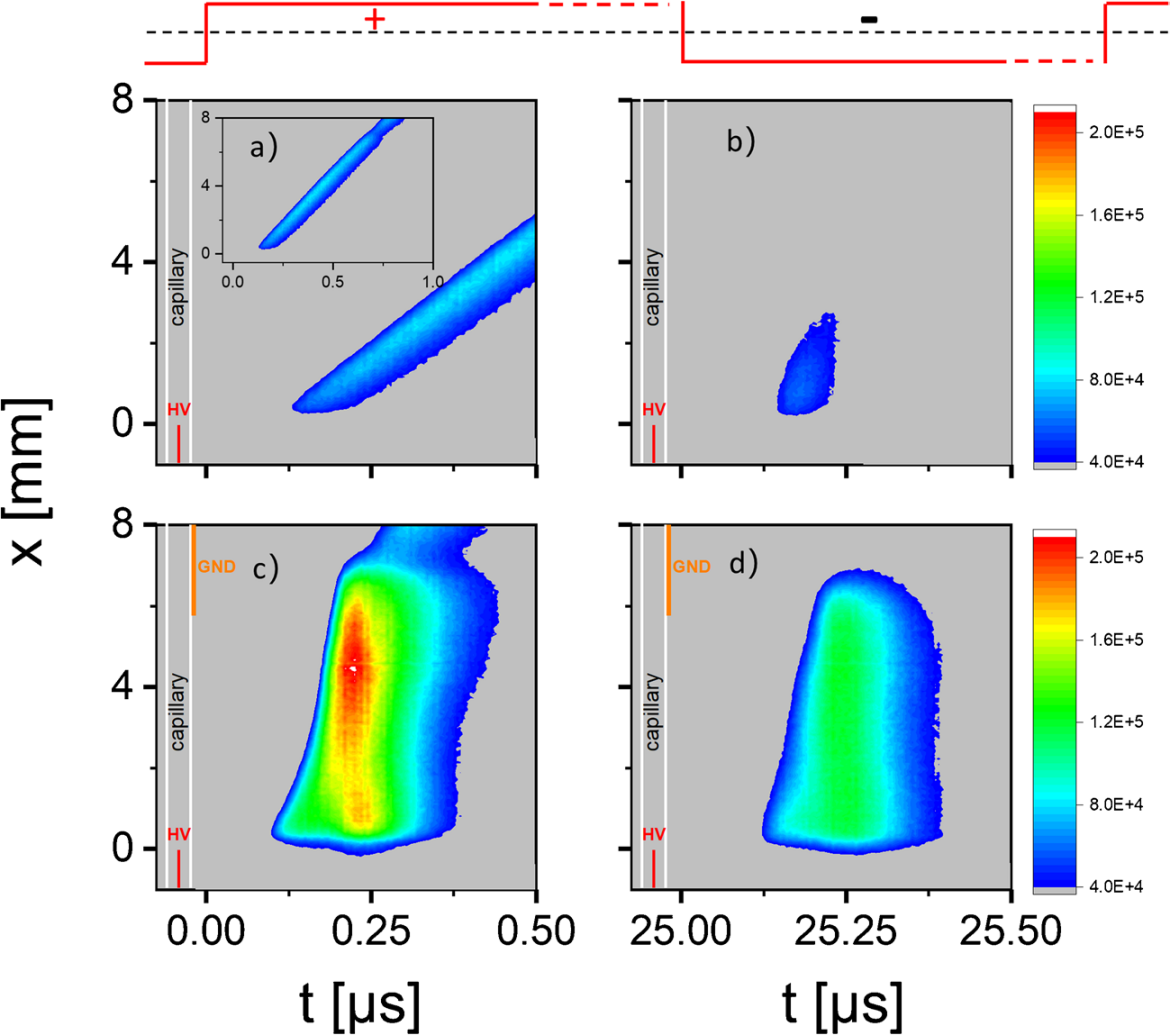
Emission color plots for N_2_^+^ 391 nm in a LµTP in **a** for the positive half cycle and in **b** for the negative. In the lower plots, a small copper sheet is touching the LµTP 5.5 cm away from the electrode, again on the left **c** for the positive half cycle and on the right **d** for the negative. He is fed as the discharge gas with a flow rate of 50 mL min^*−*1^. The applied voltage is 2.5 kV

Figure 4 shows the emission color plots for N_2_^+^ 391 nm in (a) and (b) without the copper metal sheet and in (c) and (d) with it. The emission intensities for all cases were adjusted on the same scale to make a visual comparison. It is obvious that the emission intensities of N_2_^+^ 391 nm with a grounded metal sheet, not only in the positive half cycle but also in the negative half cycle, are higher than that without a metal sheet. This indicates that more N_2_^+^ ions are generated, which can therefore produce a higher potential on the glass wall.

In Figure S2, emission plots for He 706 nm are presented. Plots (c) and (d) show the behavior of a rising and falling part of a dielectric barrier discharge. The copper metal sheet acts as a second electrode and leads to a second plasma propagating back from the touching point to the electrode. It is more powerful than the plasma going from the electrode to the end of the capillary. Therefore, the potential forming outside the capillary is stronger and leads to higher ion intensities. Additionally, the potential outside decreases rapidly with distance which makes it necessary to be as close to the MS inlet as possible.

**Fig. 5:**
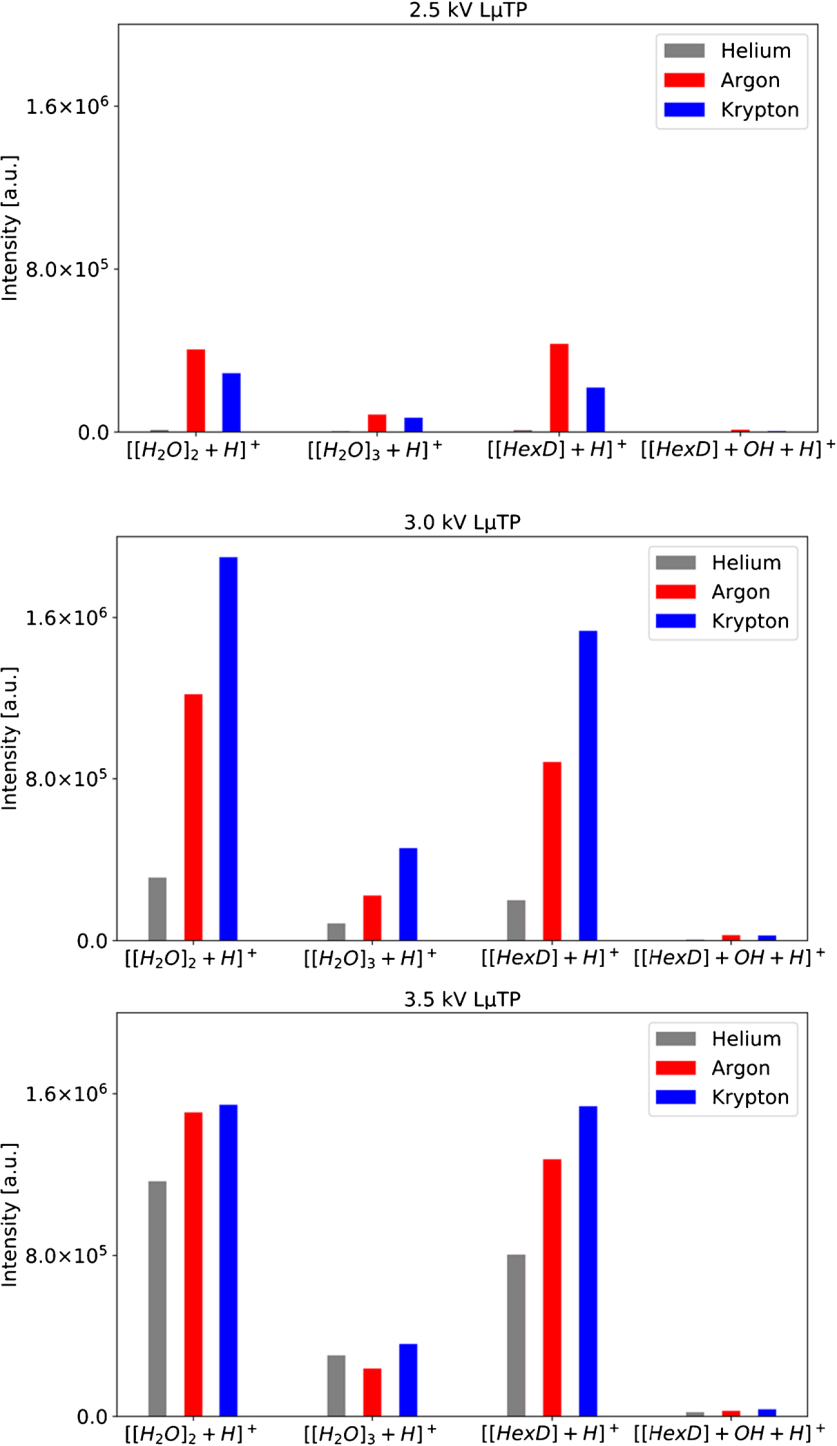
Peak intensities of [H_2_O]_2_ + H^+^, [H_2_O]_3_ + H^+^, [HexD] + H^+^, and [HexD] + OH + H^+^ obtained with He-, Ar-, and Kr-LµTP sources operated by 2.5 kV, 3.0 kV, and 3.5 kV

In addition, the ionization efficiency of LµTP operated with different noble gases was evaluated. In Figure S3, mass spectra recorded with He-, Ar-, and Kr-LµTP as an ionization source are presented. The upper spectra show the spectra without analyte with only protonated water peaks visible at [H_2_O]_2_ H^+^ at m/z 37 and [H_2_O]_3_ H^+^ at m/z 55 and in the lower part the spectra of the analyte HexD with peaks [HexD + H]^+^ at m/z 115 and [HexD + H + OH]^+^ at m/z 132. The intensities of the peaks measured with different LµTP operated with different noble gases are given in Table S2 and are shown in Fig. 5. Increasing the applied voltage has a great influence on the number of generated ions, which affects the transient potential and ultimately on the ionization efficiency. With an increase from 2.5 to 3.0 kV, the signal increases nearly fivefold, whereas with the FµTP source, the increase was only up to 18%. If the voltage of the LµTP is increased further from 3.0 to 3.5 kV, an increase in efficiency of up to 50% can be achieved, and an even smaller increase in efficiency can be expected with a higher voltage.

In Table S2, the fields with the highest intensities for the respective applied voltages are highlighted in color. For voltages of 2.5 kV, 3.0 kV, and 3.5 kV, the intensities achieved with the respective Ar and Kr sources are the highest, respectively. Interestingly, He performs the worst. It is possible that with an even higher voltage than 3.5 kV, the He-LµTP source could possibly achieve better than or comparable with the efficiencies of the other sources.

### First experiments with a self-made closed µ-tube plasma

**Fig. 6:**
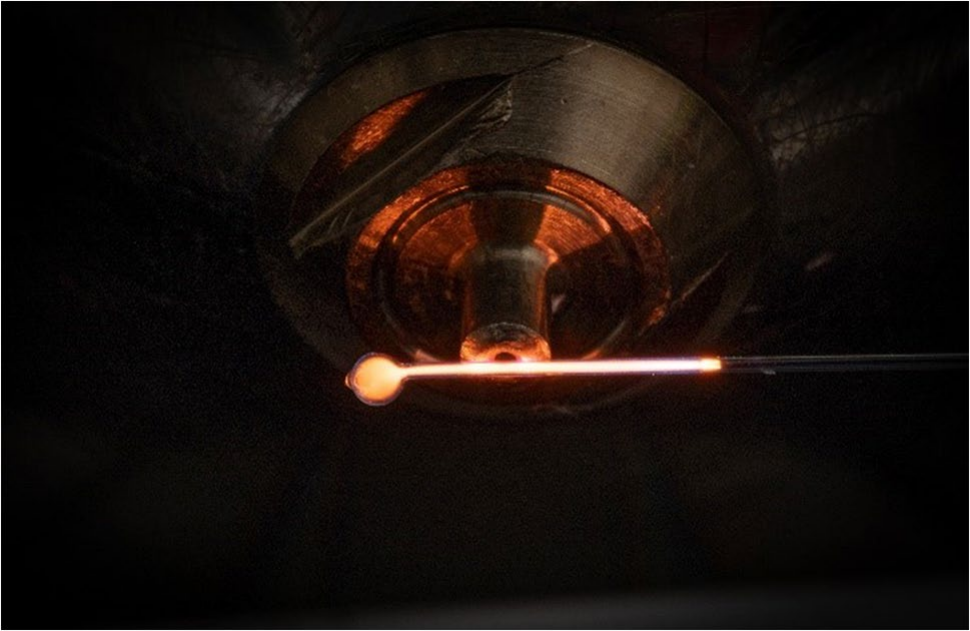
Picture of a Ne-CµTP in front of a MS inlet

As described in the experimental arrangement, CµTPs were produced with the noble gases He, Ar, and Kr. A successfully produced Ne-CµTP can be seen in Fig. 6. Neon was used because its orange color makes it easy to identify if the production process was successful. The bubble is a result of the current production process. Next to the electrode, a bluish light can be seen which is a result of N_2_ being ignited. The experimental arrangement can be compared to that of the LµTP. However, the distances between the MS inlet and the end of the electrode differed. While this distance was 10 mm for the LµTP, it was only 3–5 mm for the CµTP due to the small dimensions of only 10–15 mm. Shortening the distance between the electrode and MS inlet can lead to higher intensities of the corresponding spectra. The measured spectra for two different applied voltages, here 2.5 and 3.5 kV, are in Figure S4 and the corresponding barplot in Figure S5 and the measured intensities are shown in Table 1. The colored fields again show the highest measured intensities for the CµTP operated at 2.5 and 3.5 kV. The Ar-CµTP is the most efficient for both voltages. But the ionization efficiencies of all CµTPs are increasing with an increase of the applied voltage. Unfortunately, the amplitude of the homemade power supply used is 3.5 kV. Nevertheless, the potential of CµTP serving as a soft ionization source was initially demonstrated.

**Table 1:**
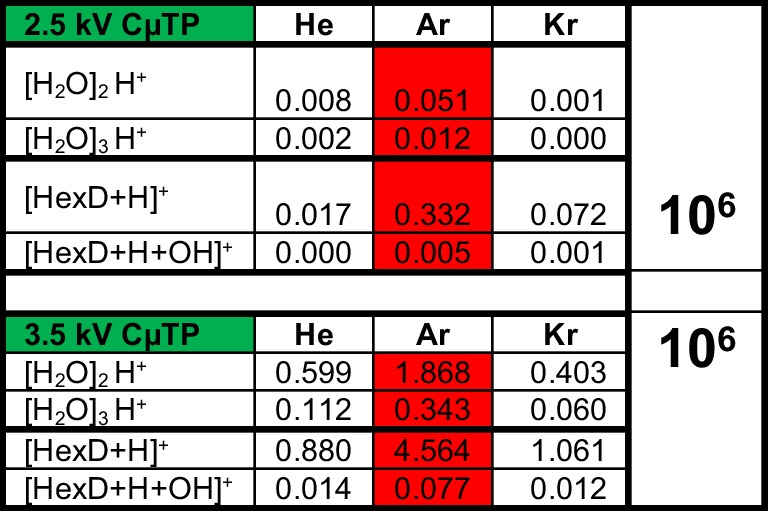
Peak intensities of [H_2_O]_2_ + H^+^, [H_2_O]_3_ + H^+^, [HexD] + H^+^, and [HexD] + OH + H^+^ when He-, Ar-, and Kr-CµTP sources operated by 2.5 kV and 3.5 kV are applied

### Efficiency comparison of FµTP, LµTP, and CµTP

In order to discuss and understand the ionization mechanism, the ionization efficiency of FµTPs, LµTPs, and CµTPs is compared, which is shown in Table 2. These are the measurements with the highest voltages applied in each case. In the case of FµTP 3 kV, since with higher voltages, no significant increase in efficiency can be expected. The other two sub-tables give information about the ionization efficiency, where there is no direct contact of excited or ionized species of the plasma gas with those of the ambient air. Here, excitation by mechanisms such as charge transfer, Penning ionization, or excitation transfer between reactive species of the plasma and the ambient air is not possible. Ionization is initiated here exclusively by a temporally and spatially variable positive potential at the outer wall of the capillary. Electrons are accelerated due to a potential difference between the surface of the glass capillary and its surroundings, so that ions are generated by collision of electrons with species of the ambient air. As indicated in the sub-tables, the LµTPs and the CµTPs, where there is no contact of the plasma species with those of the ambient air, were operated at a higher voltage than that of the FµTPs. The reason for this is the dielectric barrier in the form of the glass capillary wall, where a higher voltage is required than that necessary to form a transient potential at the end of the open capillary of the FµTPs. As mentioned above, a further increase of the voltage at the FµTPs leads only to small increases of the intensities, while a further increase of the applied voltages at the LµTPs and the CµTPs could well lead to further increases of the intensities. The colored fields show the highest intensities of each sub-table. In the case of the FµTP, the one with Kr as the plasma gas has reached the highest intensities with respect to analyte ionization and the one with He with respect to water ionization. While water ionization using all three FµTPs gave a similar result, with ionization of water by a He-FµTP appearing slightly more effective, the intensities of the analyte signals for the Ar- and Kr-FµTP set themselves apart from the others. In the case of the LµTP, the ionization of both the analyte and the water is the highest for the Kr-LµTP, while in the case of the CµTP, the ionization efficiency of the Ar-CµTP appears to be the highest.

**Table 2:**
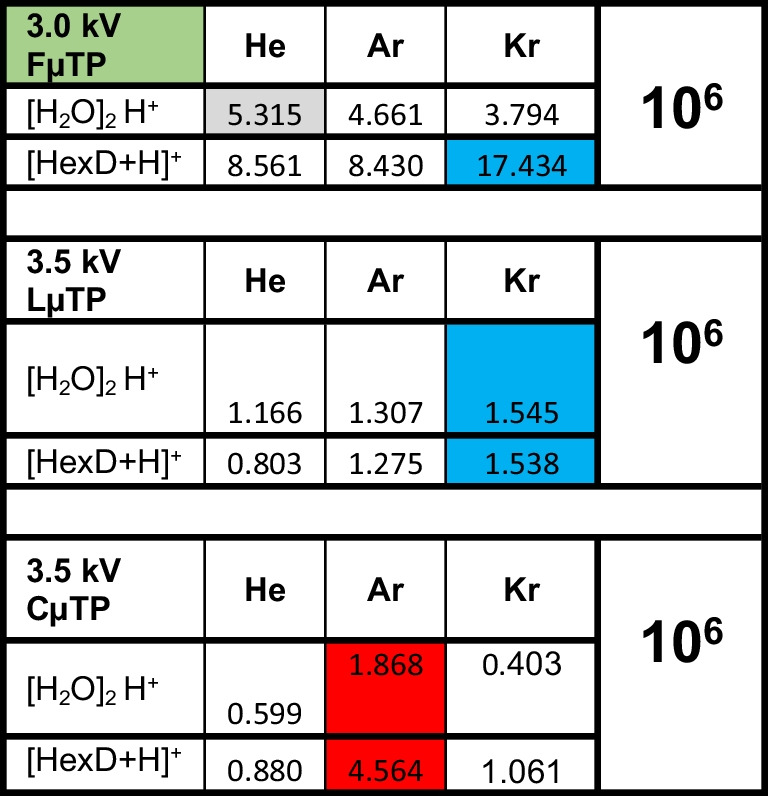
Comparison of [H_2_O]_2_H^+^ and HexD + H]^+^ peak intensities measured in FµTPs, LµTPs, and CµTPs sources operated by 3.0 kV, 3.5 kV, and 3.5 kV, respectively

Although the high intensities for Kr-FµTP cannot be explained, it is ruled out for use as ionization sources anyway because the cost of operation is too high. In the case of CµTP, filling a case with Ar is optimal for both detection strength and fabrication cost reasons. Compared to both the He-FµTP, which is used in the majority of all analytical measurements, and the Ar-FµTP, with which similar detection strengths can be achieved as with the He-FµTP, the detection strength of the CµTP is about a factor of 2 worse.

## Conclusions

It was found that soft ionization was not related to either charge transfer or Penning ionization of species of air with species of plasma, as here the ionization efficiency of an open plasma was compared with that of plasmas with the wall of a glass capillary between the plasma volume and the ambient air. In [8], it could be shown that photoionization plays a minor role in the ionization mechanism. This possibility could be excluded completely since the glass wall of the capillary does not transmit radiation below 300 nm anyway. These are rather basic measurements showing that a positive potential is formed at the outer surface of the capillary for a limited time and must lead to electrons being accelerated towards the capillary, thus forming ions by collision with species of the ambient air, which are responsible for the soft ionization. A similar mechanism also applies in the case of the plasma of an FµTP open to the ambient air. Here, temporally and spatially limited ion clouds are generated, where the electrons perform the ionization of the outer air in the same way. In addition, the charge on the glass at the end of the capillary could enhance this effect. Therefore, it is possible to perform the same mechanism at least with any noble gas. It is not the energy of the particular ion state of the noble gas that is decisive, but the number of ions that form an ion cloud. The more ions are in an ion cloud for a short time, the higher is the potential. With the same mass flow through the plasma capillary, the same number of atoms moves through the capillary for each of the noble gases, and thus the same number of ions for one gas and voltage can be expected at the end of the capillary of the FµTP. In the case of the LµTP and the CµTP, the same is true for the number of ions attached to the inner wall of the capillary for a short time and thus the positive potential, which is created by polarization of the glass layer on the outside of the capillary.

In addition to the basic research, an ionization source was invented that enables effective ionization comparable to that of the FµTP without ensuring a continuous gas flow.

## Supplementary Information

Below is the link to the electronic supplementary material.

Supplementary file1 (DOCX 5633 KB)
