# Supplementary material for: Soft ionization mechanisms in flexible µ-tube plasma—from FµTP to closed µ-tube plasma
Source: Anal Bioanal Chem. 2024 Jul 3;416(22):4919–27. doi: 10.1007/s00216-024-05420-8 (PMC11496370; doi:10.1007/s00216-024-05420-8)

**Supplementary to**

**Soft Ionization Mechanisms in Flexible µ-Tube Plasma - From FµTP to Closed µ-Tube Plasma**

Luisa Speicher^a^, Hao Song^a^, Norman Ahlmann^a^, Daniel Foest^a^, Simon Höving^a^, Sebastian Brandt^a^, Guanghui Niu^b^, Joachim Franzke^a^*, Caiyan Tian^a^*

^a^Leibniz-Institute for Analytical Sciences – ISAS – eV., Bunsen-Kirchhoff-Straße 11, 44139 Dortmund, Germany

^b^Laboratory of Inorganic Chemistry, Department of Chemistry and Applied Biosciences, ETH Zurich, Vladimir-Prelog-Weg 1, 8093 Zurich, Switzerland

***** Corresponding author: Email: [caiyan.tian@isas.de](mailto:caiyan.tian@isas.de), and [joachim.franzke@isas.de](mailto:joachim.franzke@isas.de), Phone: +49 (0)2 31.13 92-284, and +49 (0)2 31.13 92-174

**Table S1.** Peak intensities of [H_2_O]_2_H^+^, [H_2_O]_3_H^+^, [HexD+H]^+^ and [HexD+OH+H]^+^ when He-, Ar- and Kr-**FµTP** sources operated by 2.5 kV and 3.0 kV are applied. Data for Xe are taken from part 2 but are not used for the mean values.

| **2.5 kV FµTP** | Helium | Argon | Krypton | mean | 10^6^ |
| --- | --- | --- | --- | --- | --- |
| [H_2_O]_2_ H^+^ | 4.882 | 4.809 | 3.232 | 4.308 |  |
| [H_2_O]_3_ H^+^ | 1.258 | 1.203 | 0.732 | 1.064 |  |
| [HexD+H]^+^ | 8.159 | 7.269 | 13.725 | 9.718 |  |
| [HexD+H+OH]^+^ | 0.444 | 0.488 | 0.460 | 0.464 |  |
|  | | | | | |
| **3.0 kV FµTP** | Helium | Argon | Krypton | mean | m_3.0_/m_2.5_ |
| [H_2_O]_2_ H^+^ | 5.315 | 4.661 | 3.794 | 4.59 | 1.07 |
| [H_2_O]_3_ H^+^ | 1.345 | 1.178 | 0.850 | 1.124 | 1.06 |
| [HexD+H]^+^ | 8.562 | 8.431 | 17.434 | 11.476 | 1.18 |
| [HexD+H+OH]^+^ | 0.487 | 0.460 | 0.478 | 0.475 | 1.02 |

**
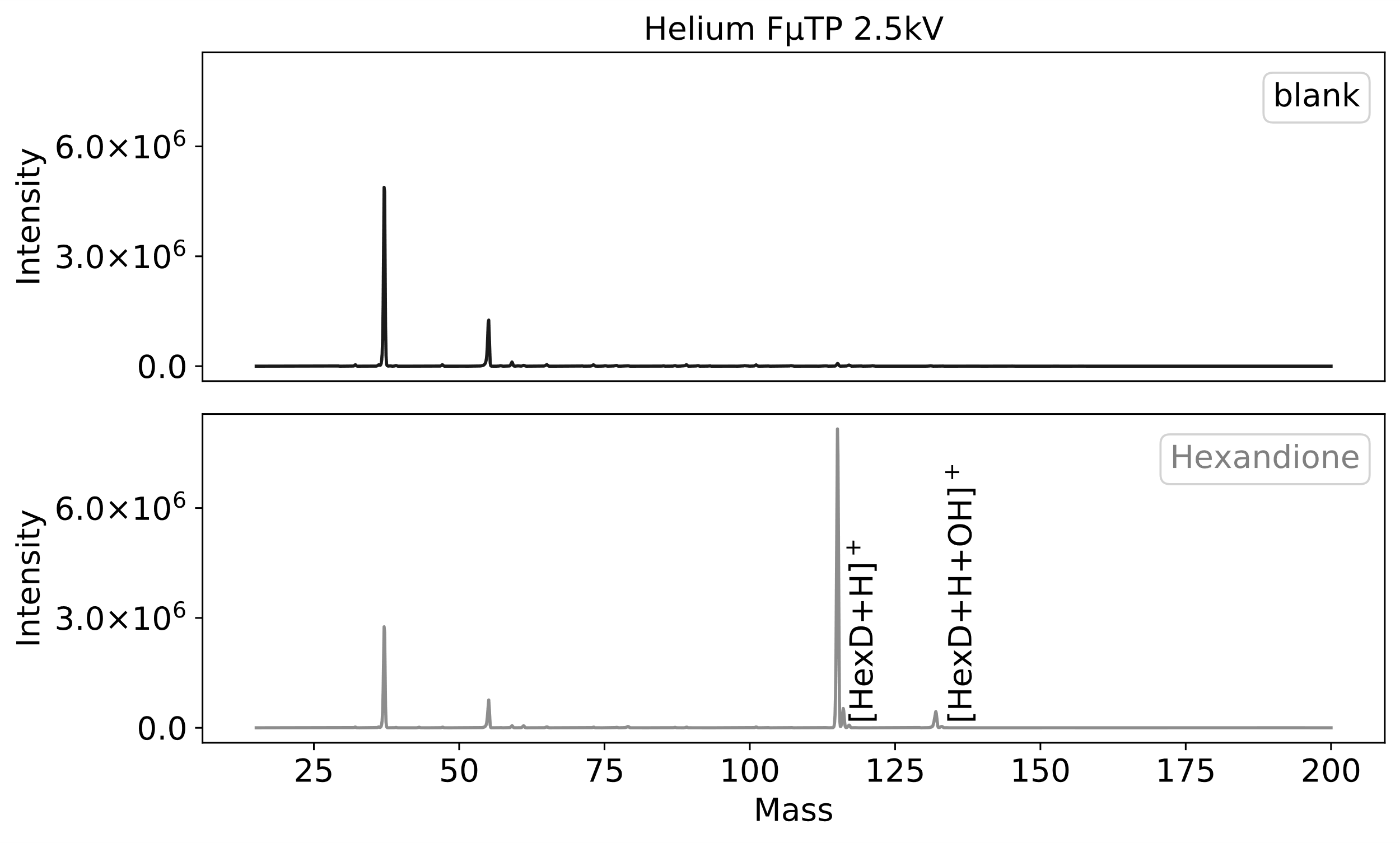

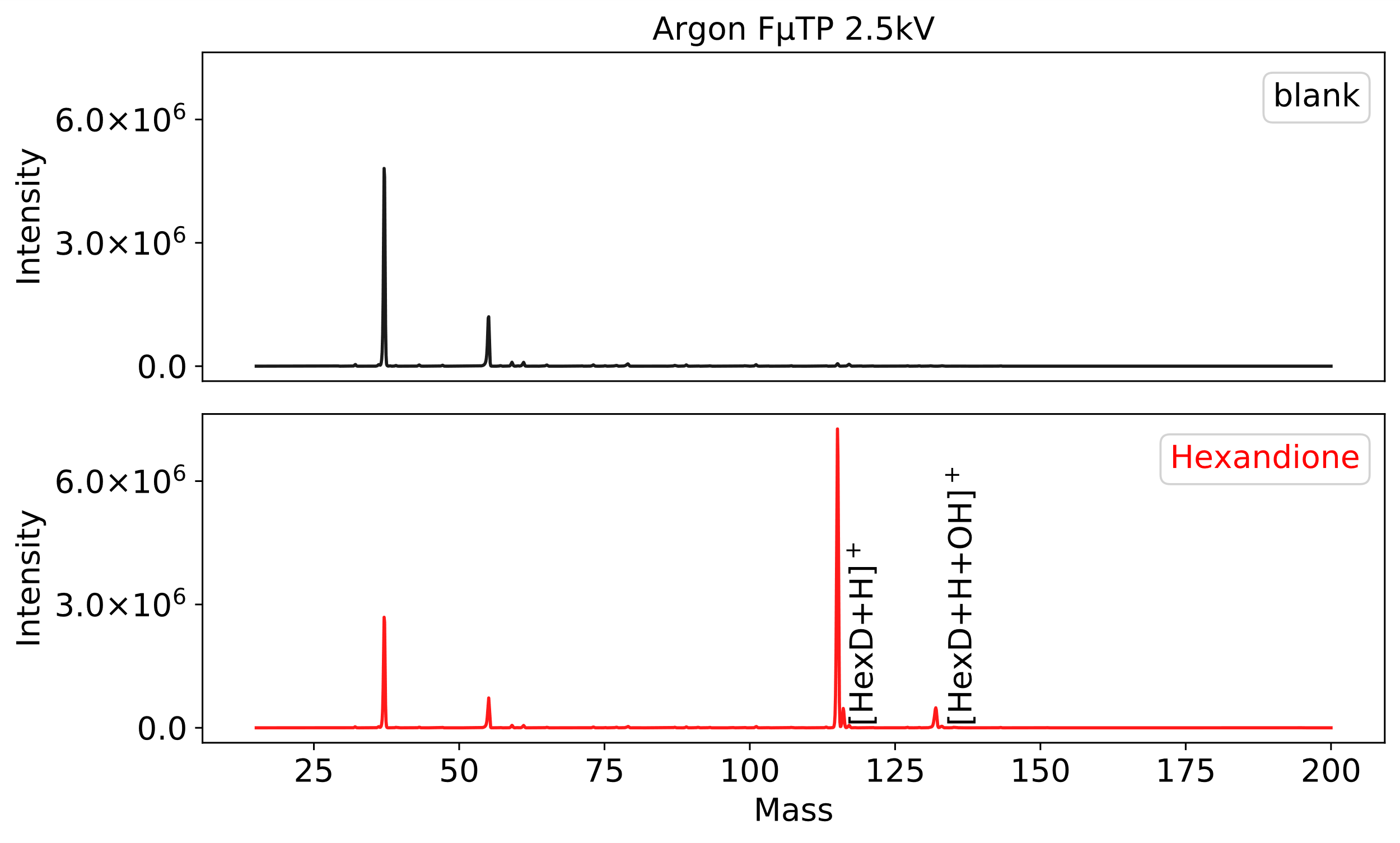

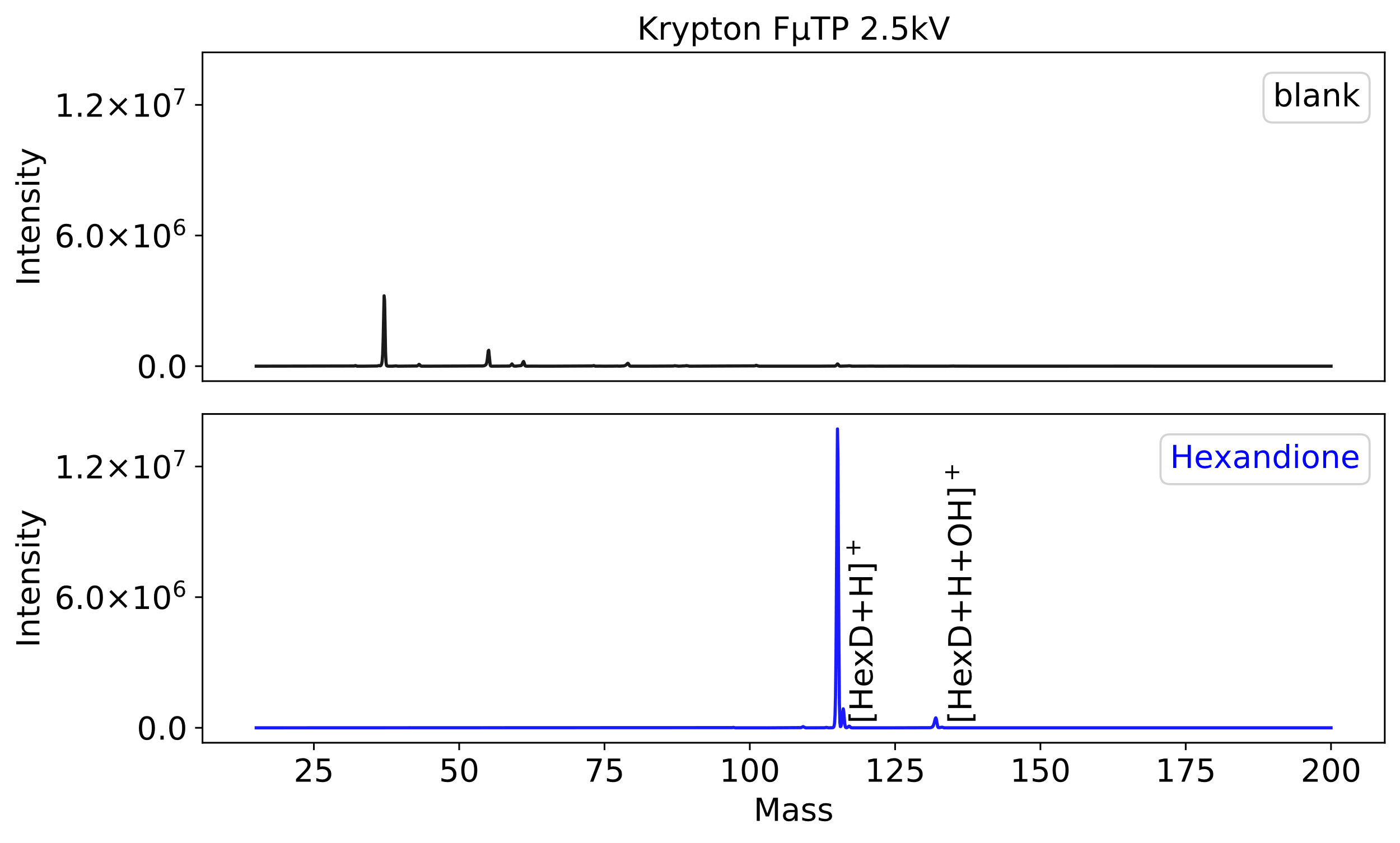

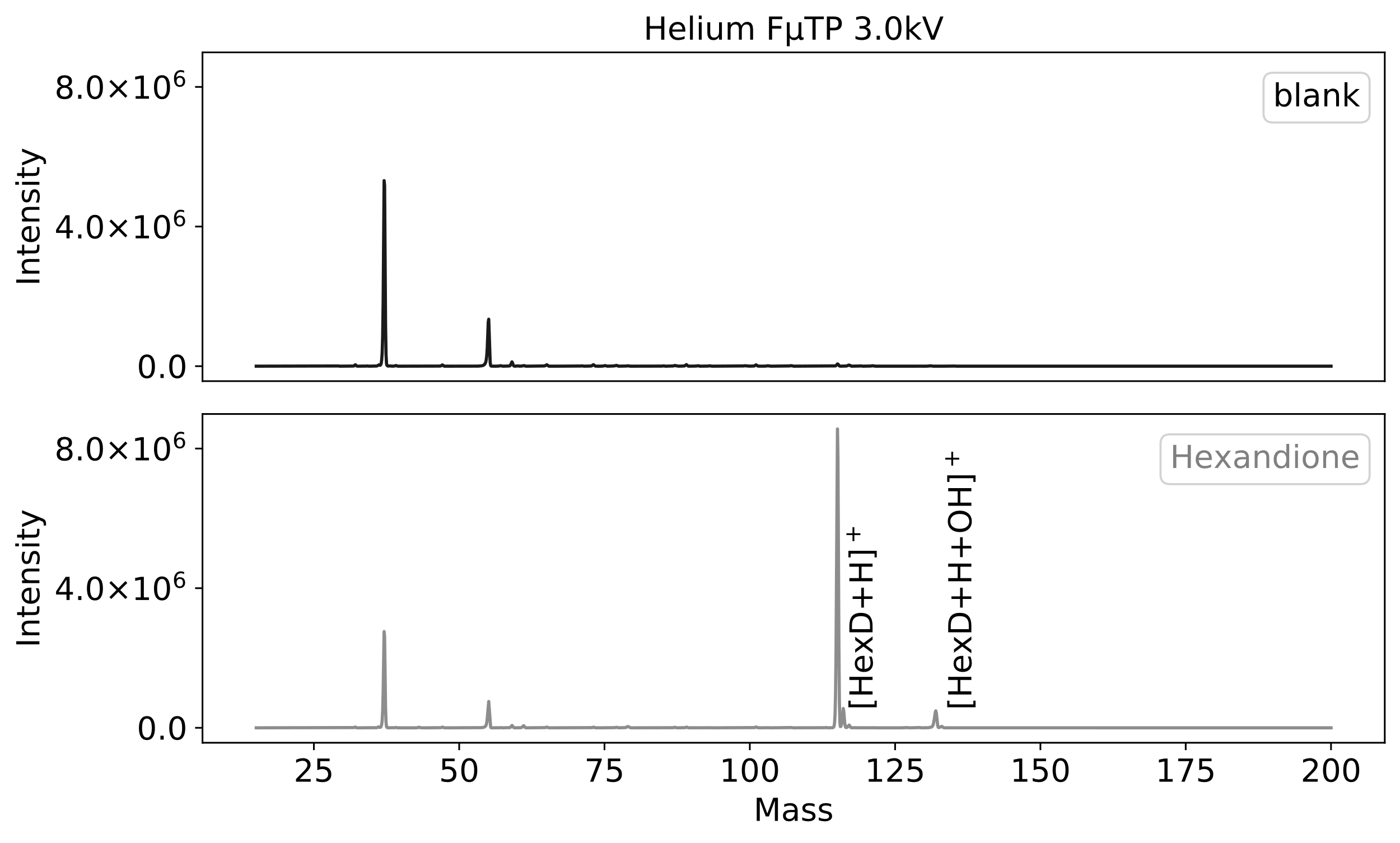

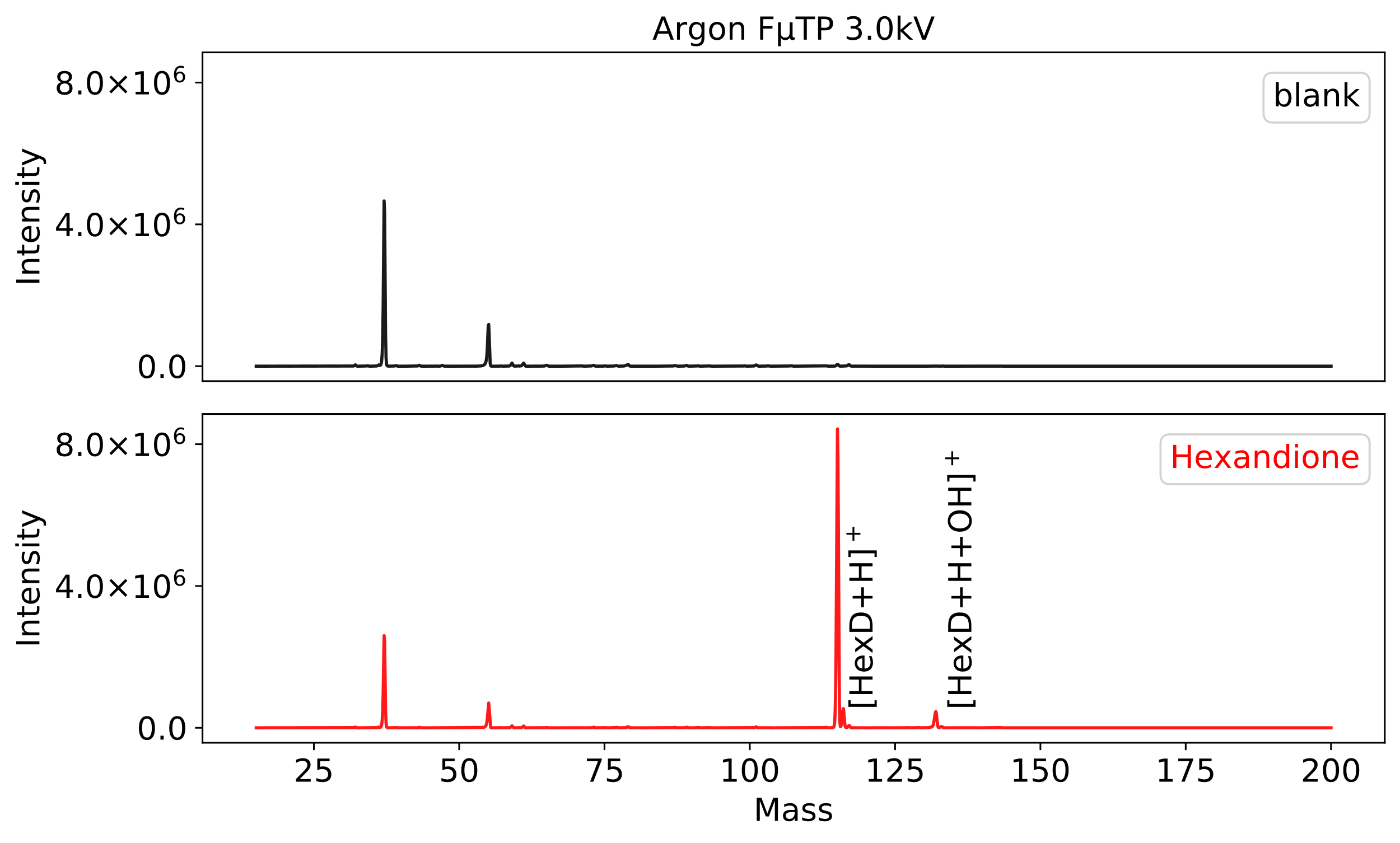

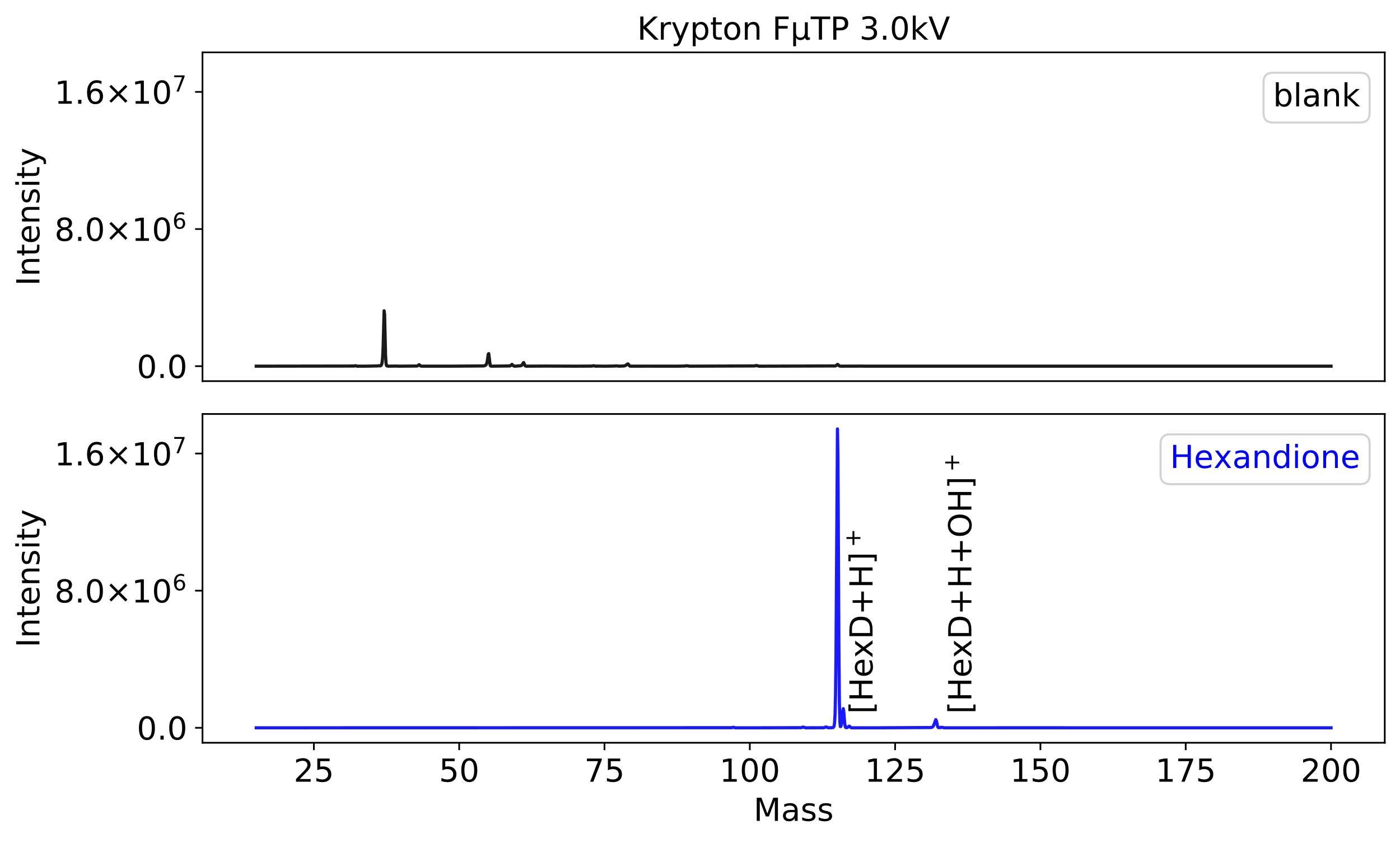
**

**Figure S1.** Blank- and analyte-spectra of Hexandion when the ionisation sources He-, Ar- and Kr-FµTP are operated by 2.5 kV and 3.0 kV.


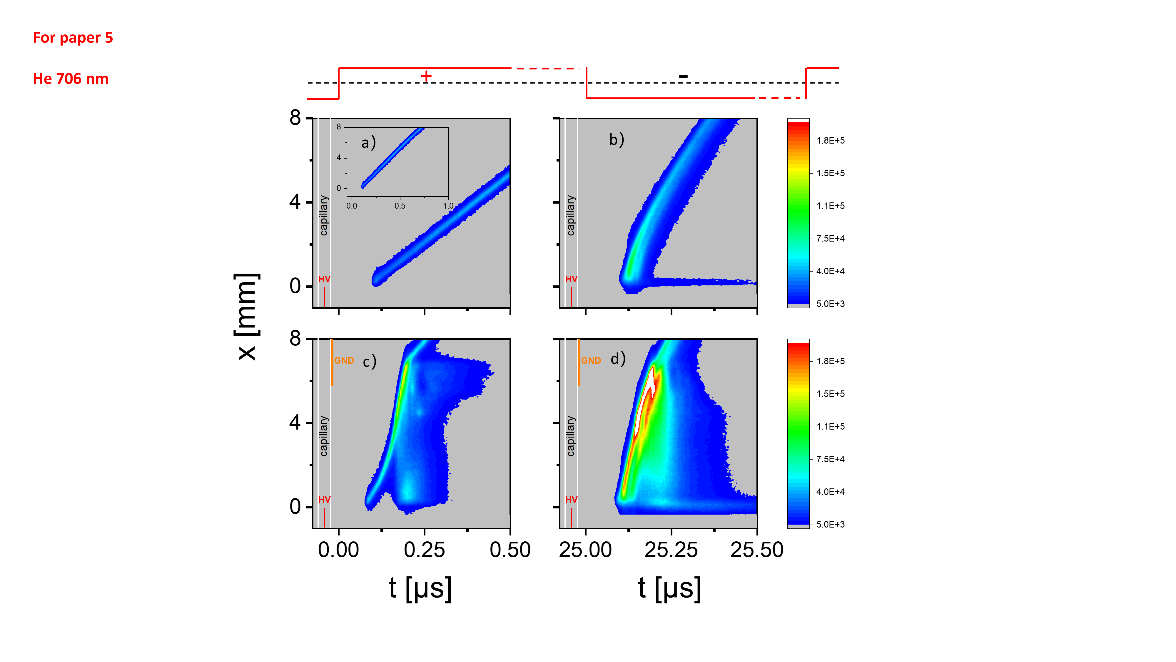


**Figure S2**. Emission color plots for He 706 nm in a LµTP in a) for the positive half cycle and in b) for the negative. In the lower pictures a copper plate is touching the LµTP 5.5 cm away from the electrode, again on the left c) for the positive half cycle and on the right d) for the negative. . He is fed as discharge gas with a flow rate of 50 mL min^-1^. The applied voltage is 2.5 kV.


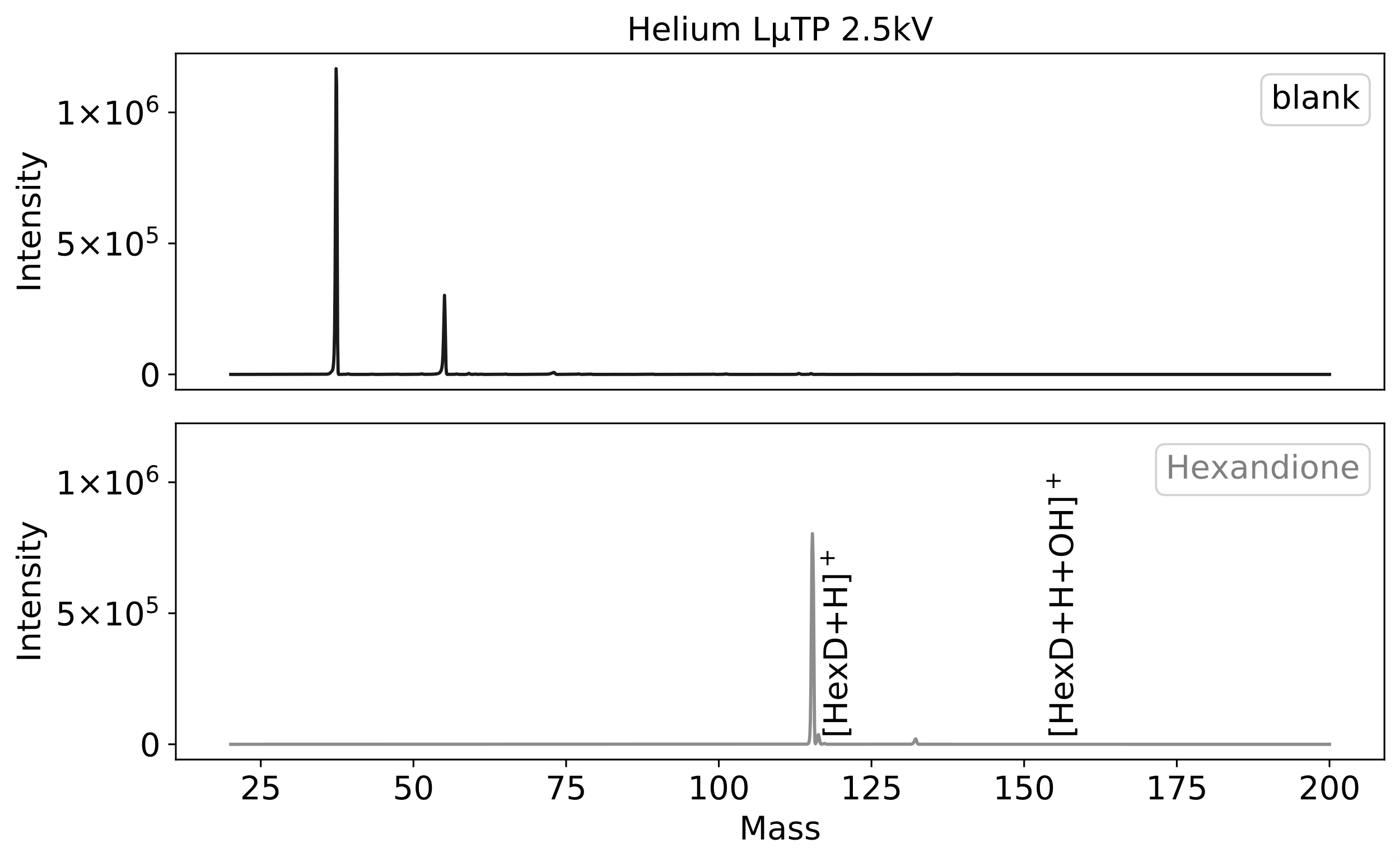

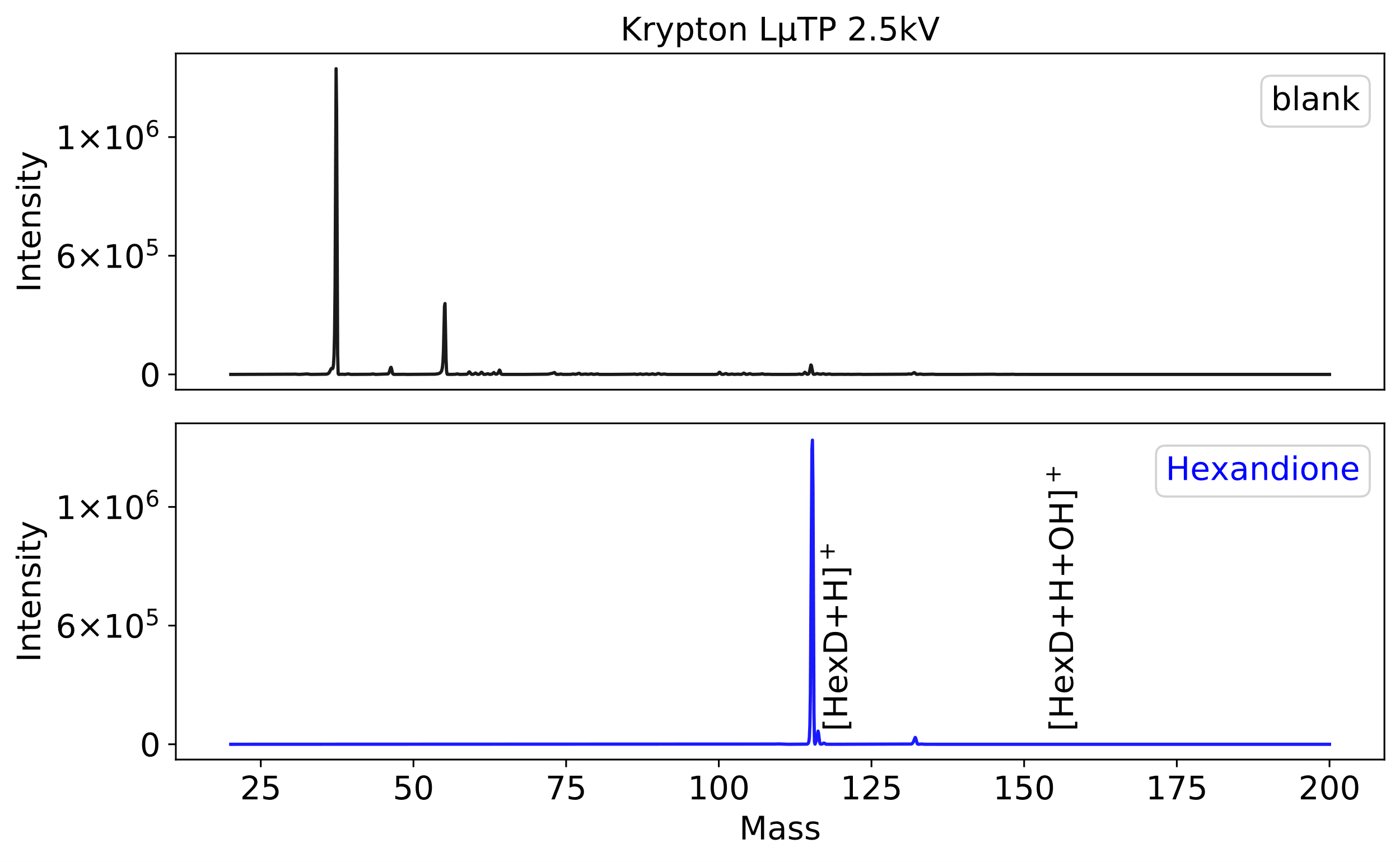

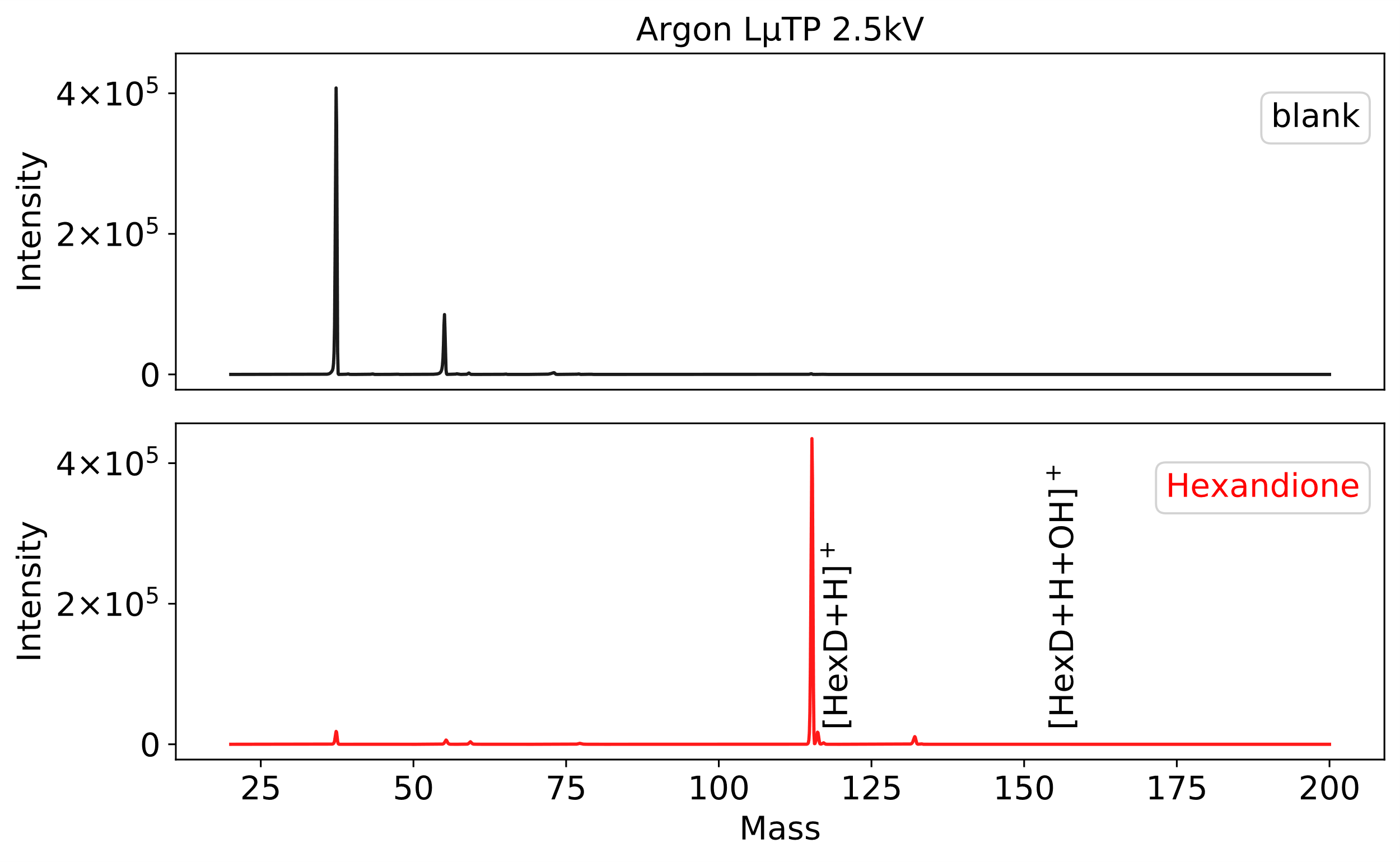

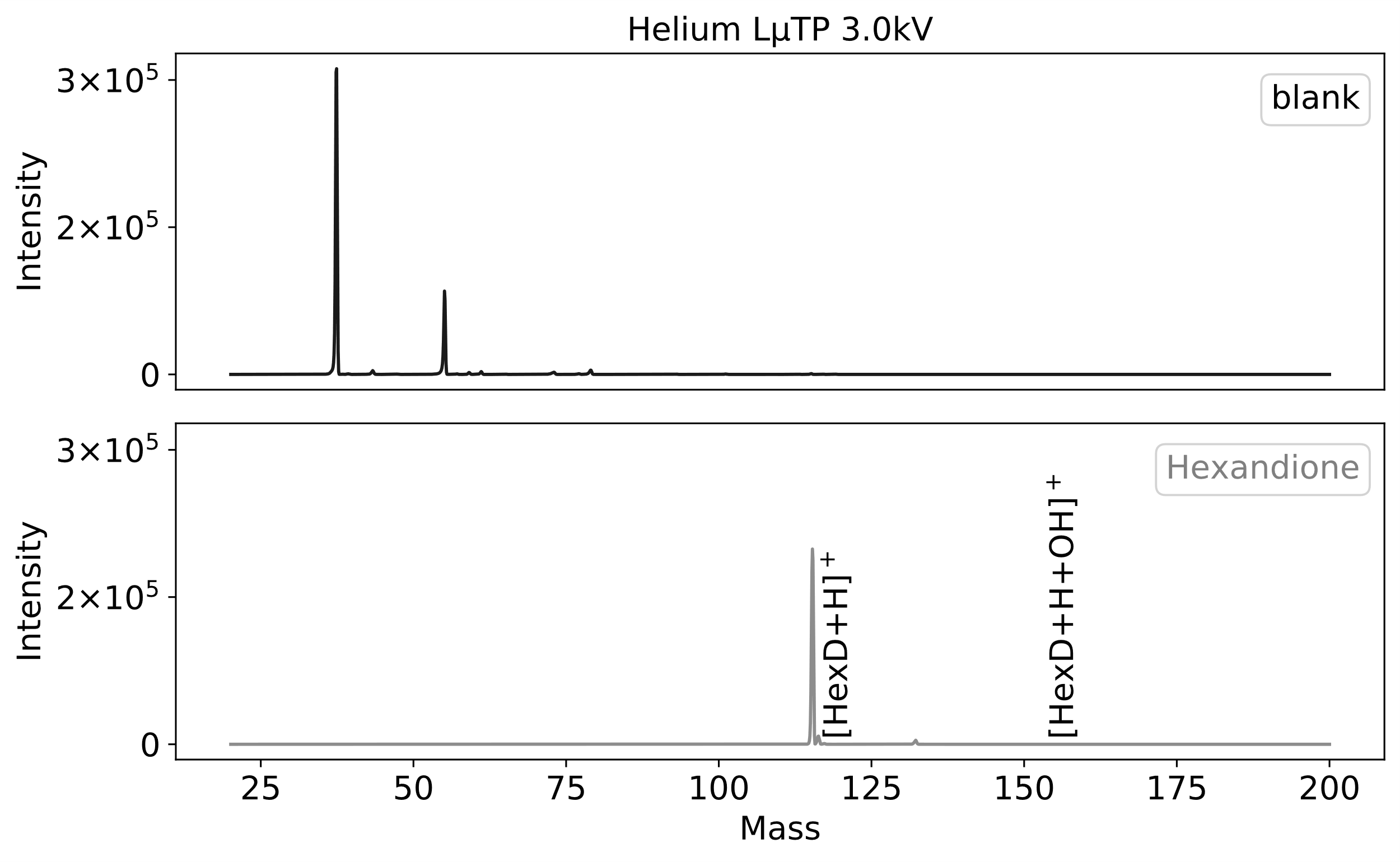

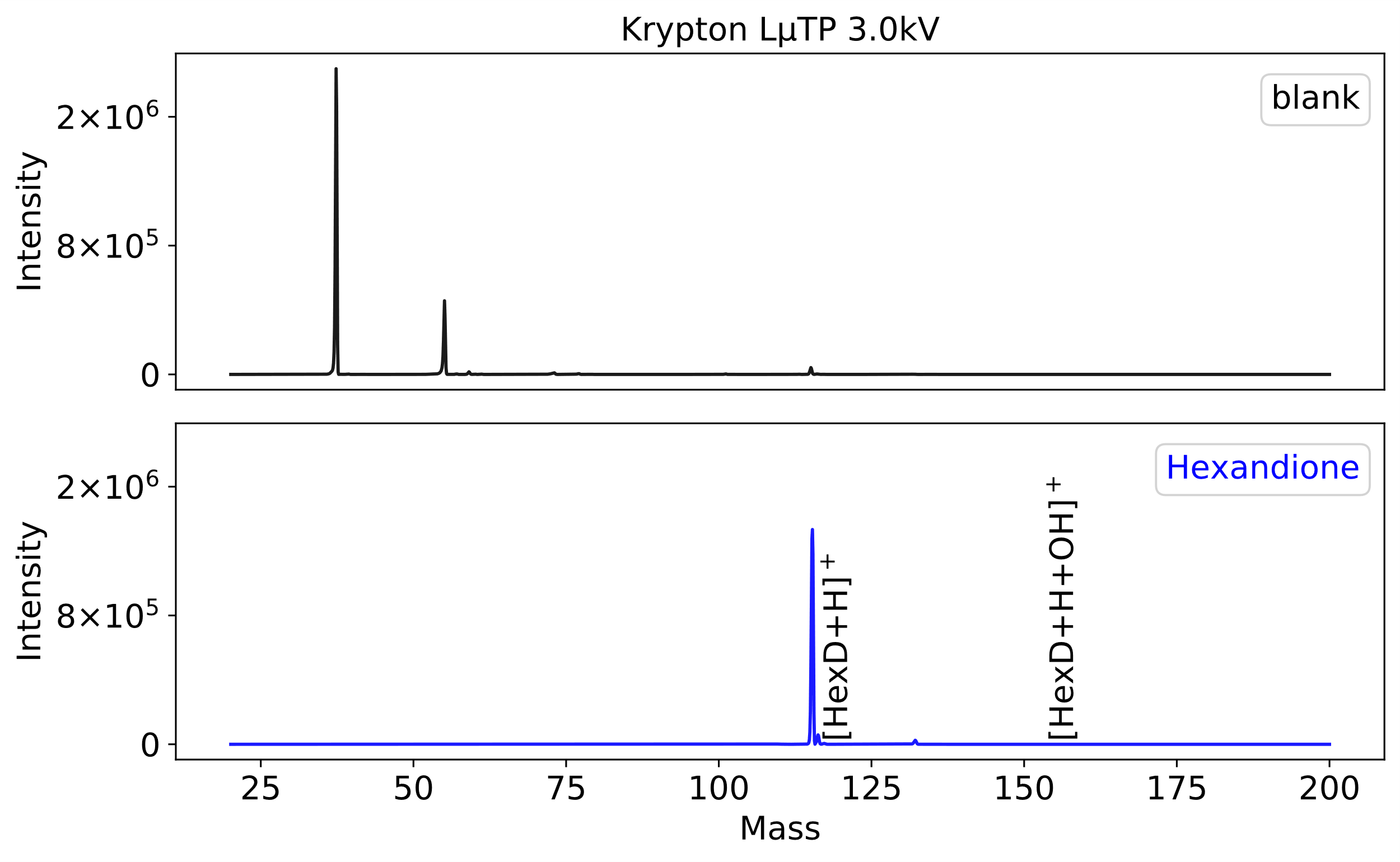

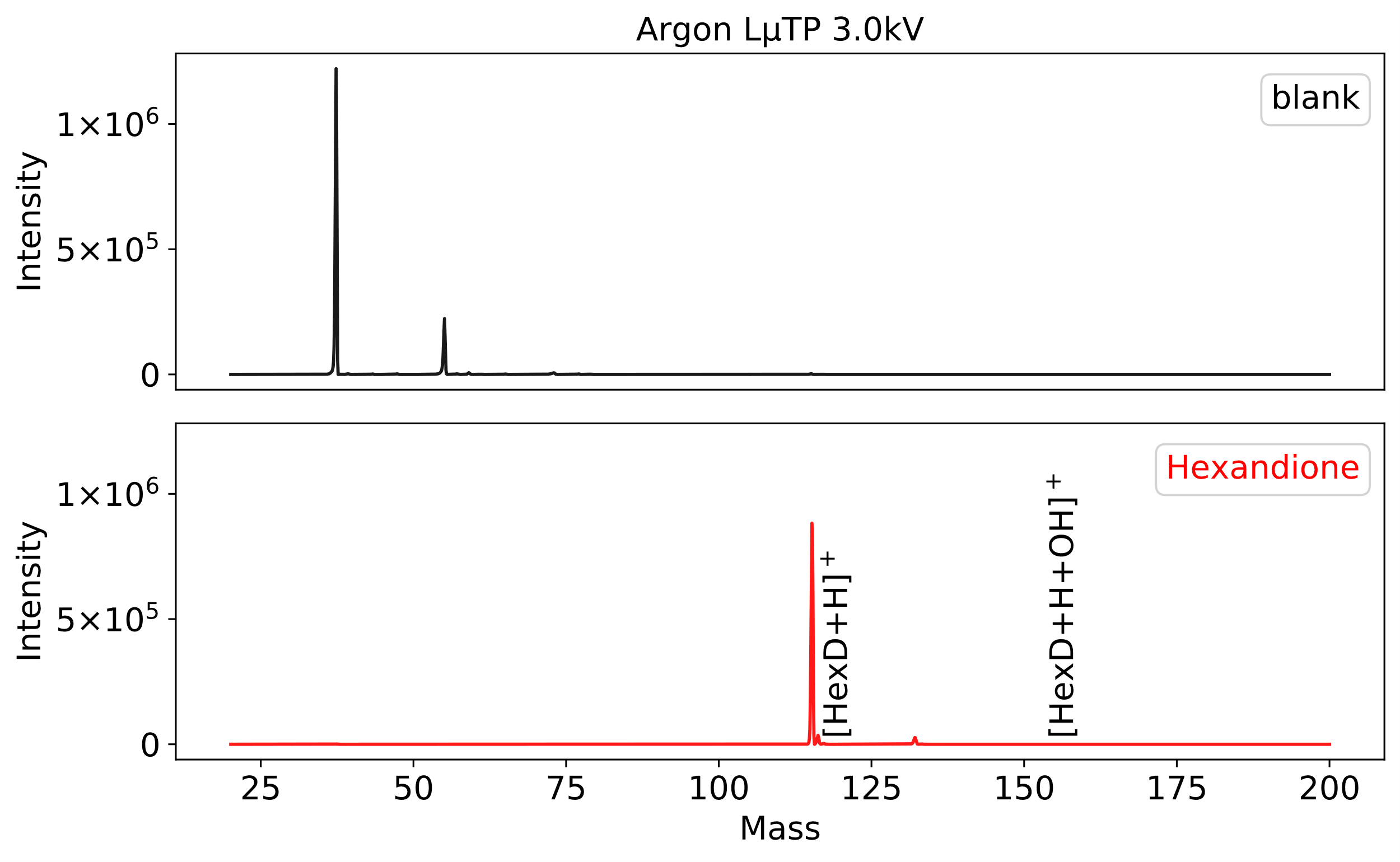

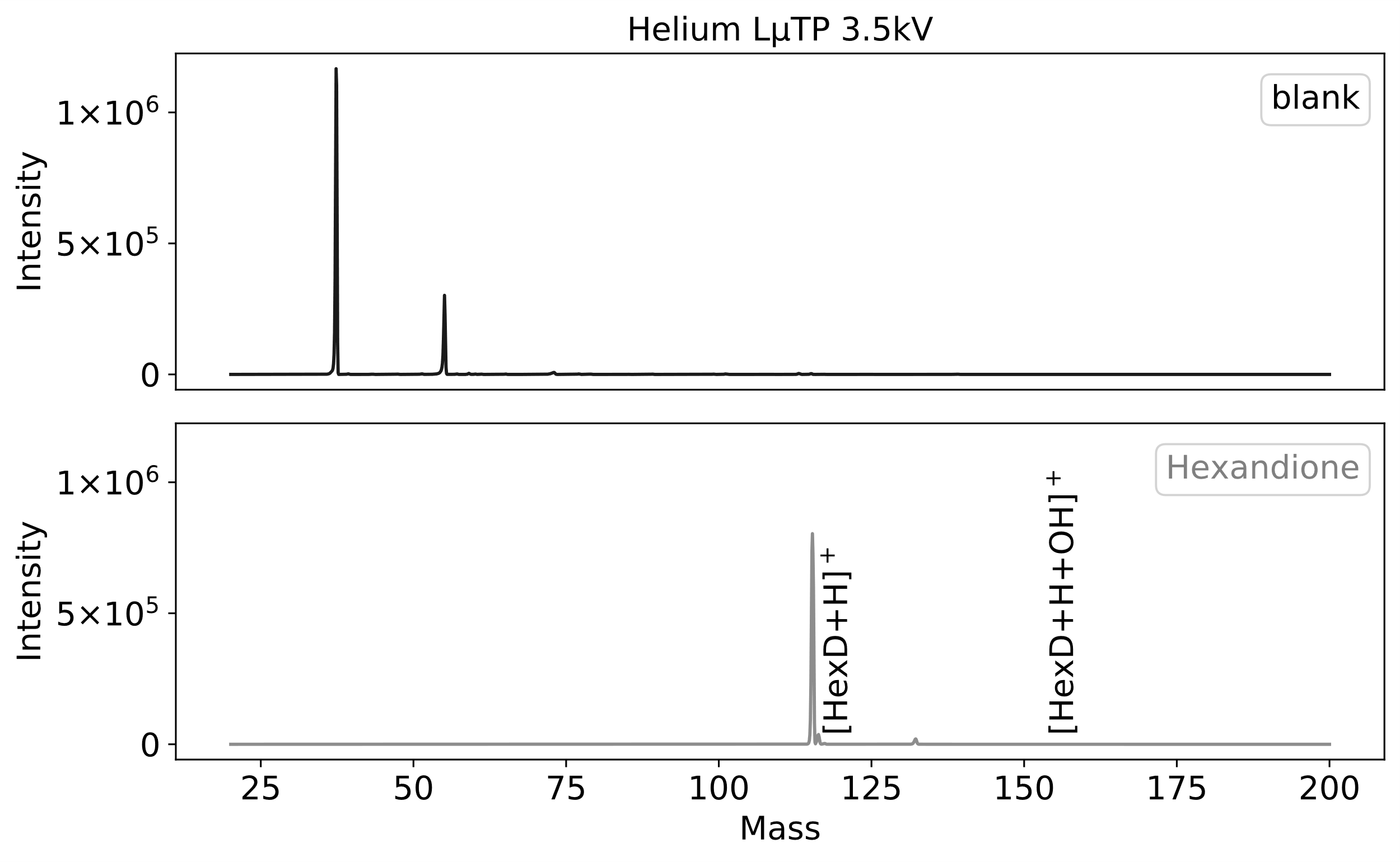

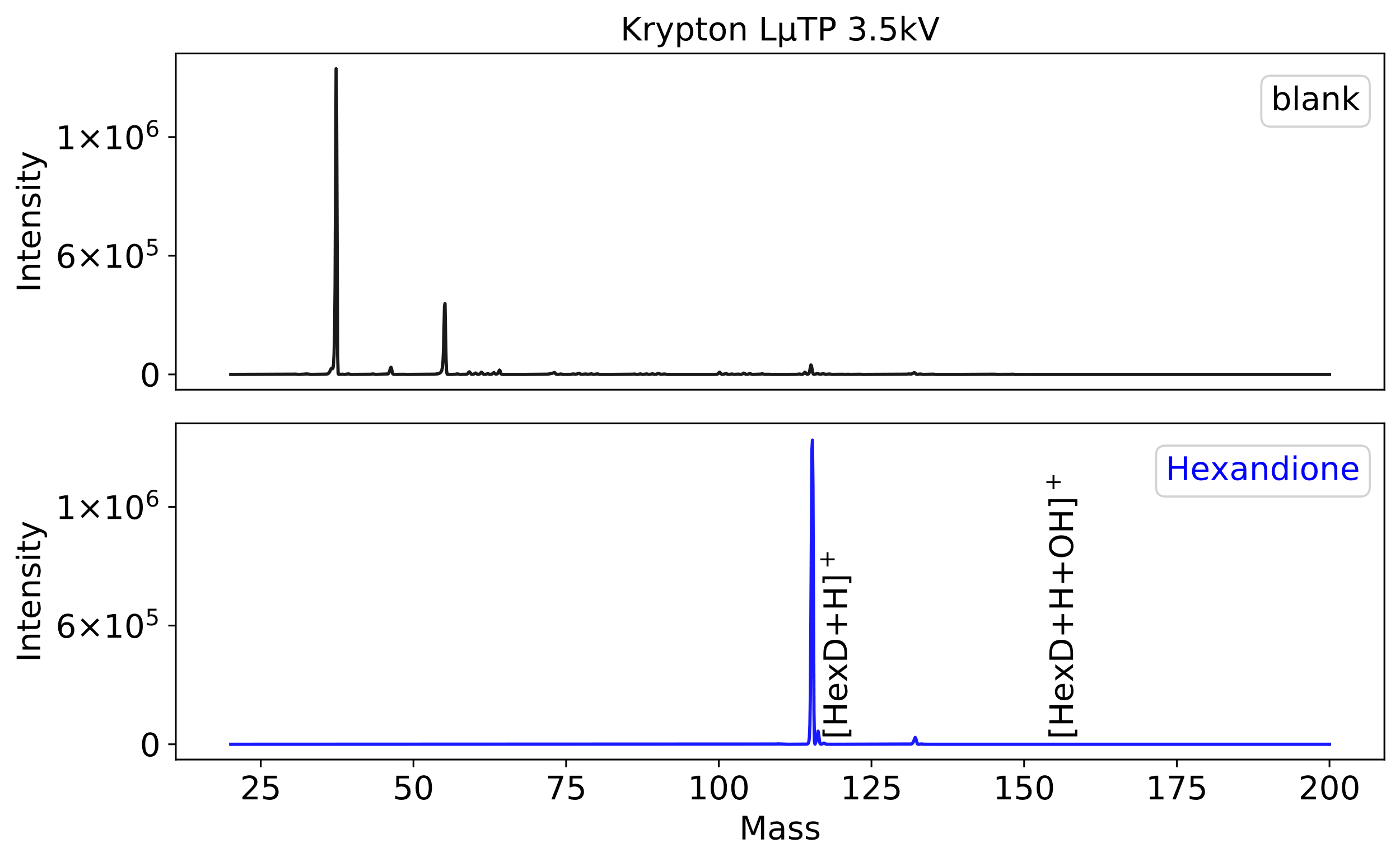

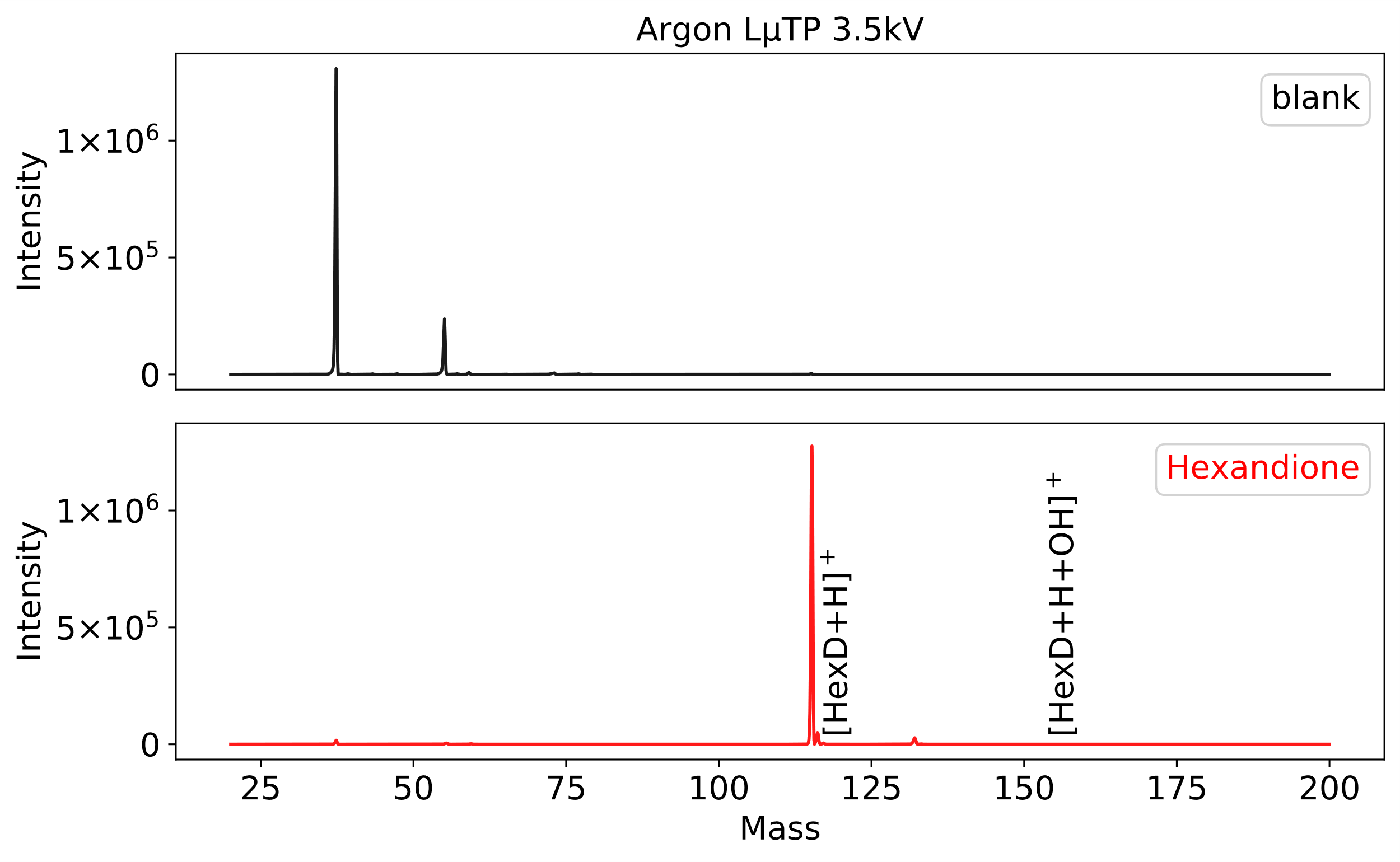


**Figure S3**. Blank-spectra and analyte spectra of Hexandion when the ionisation sources He-, Ar- and Kr-LµTP are operated 2.5 kV, 3.0 kV and 3.5 kV.

**Table S2.** Peak intensities of [H_2_O]_2_+H^+^, [H_2_O]_3_+H^+^, [HexD]+H^+^ and [HexD]+OH+H^+^ when He-, Ar- and Kr-**LµTP** sources are operated at 2.5 kV, 3.0 kV and 3.5 kV.

| **2.5 kV LµTP** | **He** | **Ar** | **Kr** | mean_2.5_ | 10^6^ | |
| --- | --- | --- | --- | --- | --- | --- |
| [H_2_O]_2_ H^+^ | 0.011 | 0.407 | 0.291 | 0.236 |  |  |
| [H_2_O]_3_ H^+^ | 0.004 | 0.085 | 0.070 | 0.053 |  |  |
| [HexD+H]^+^ | 0.009 | 0.435 | 0.221 | 0.222 |  |  |
| [HexD+H+OH]^+^ | 0.000 | 0.011 | 0.004 | 0.005 |  |  |
|  | | | | | |  |
| **3.0 kV LµTP** | **He** | **Ar** | **Kr** | mean_3.0_ | m_3.0_/m_2.5_ | 10^6^ |
| [H_2_O]_2_ H^+^ | 0.311 | 1.221 | 1.898 | 1.143 | 4.83 |  |
| [H_2_O]_3_ H^+^ | 0.085 | 0.223 | 0.457 | 0.255 | 4.81 |  |
| [HexD+H]^+^ | 0.199 | 0.883 | 1.334 | 0.805 | 3.63 |  |
| [HexD+H+OH]^+^ | 0.004 | 0.026 | 0.025 | 0.018 | 3.66 |  |
|  | | | | | |  |
| **3.5 kV LµTP** | **He** | **Ar** | **Kr** | mean_3.5_ | m_3.5_/m_3.0_ | 10^6^ |
| [H_2_O]_2_ H^+^ | 1.166 | 1.307 | 1.545 | 1.339 | 1.17 |  |
| [H_2_O]_3_ H^+^ | 0.301 | 0.237 | 0.358 | 0.299 | 1.17 |  |
| [HexD+H]^+^ | 0.804 | 1.275 | 1.538 | 1.206 | 1.50 |  |
| [HexD+H+OH]^+^ | 0.020 | 0.027 | 0.035 | 0.027 | 1.49 |  |


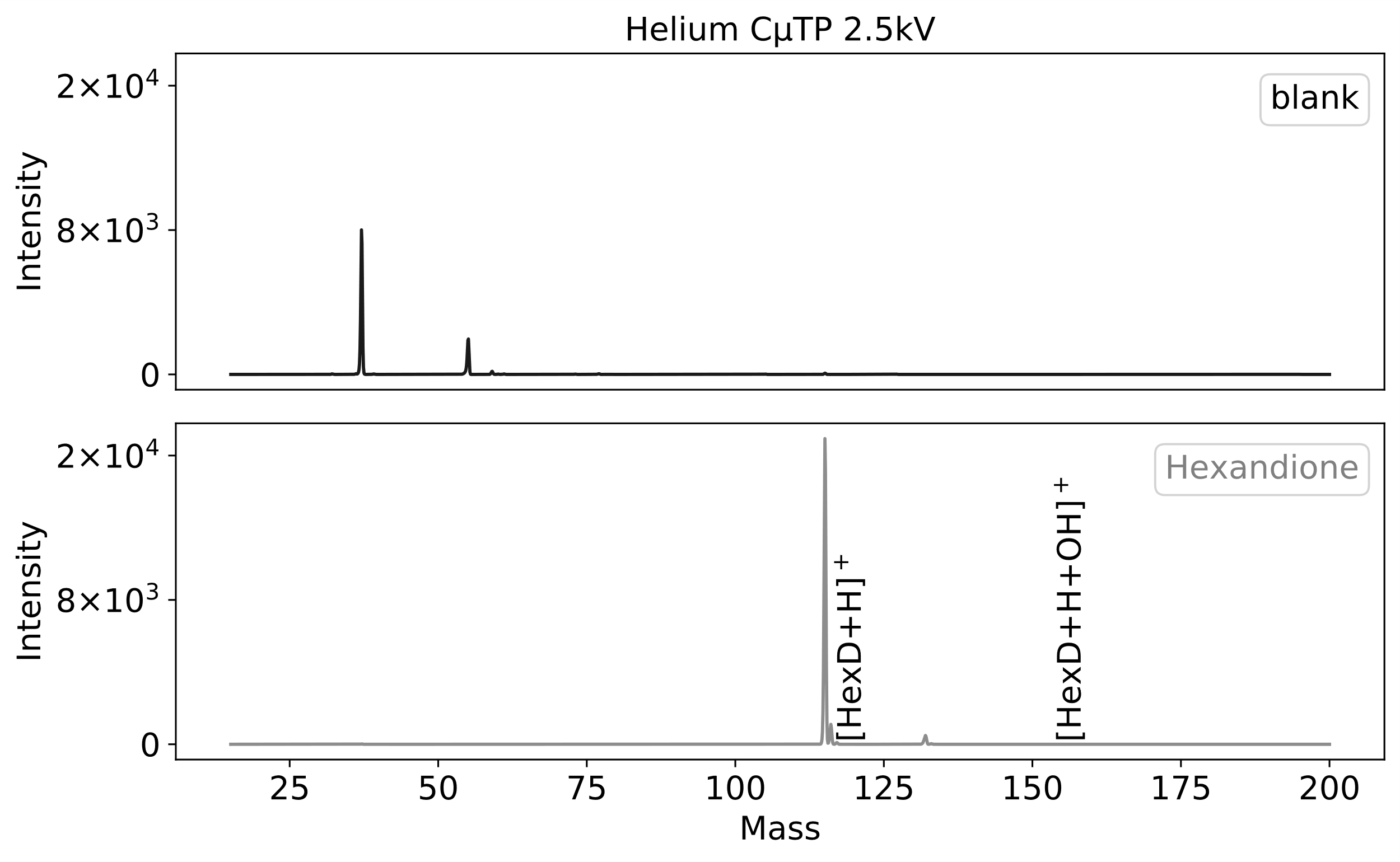

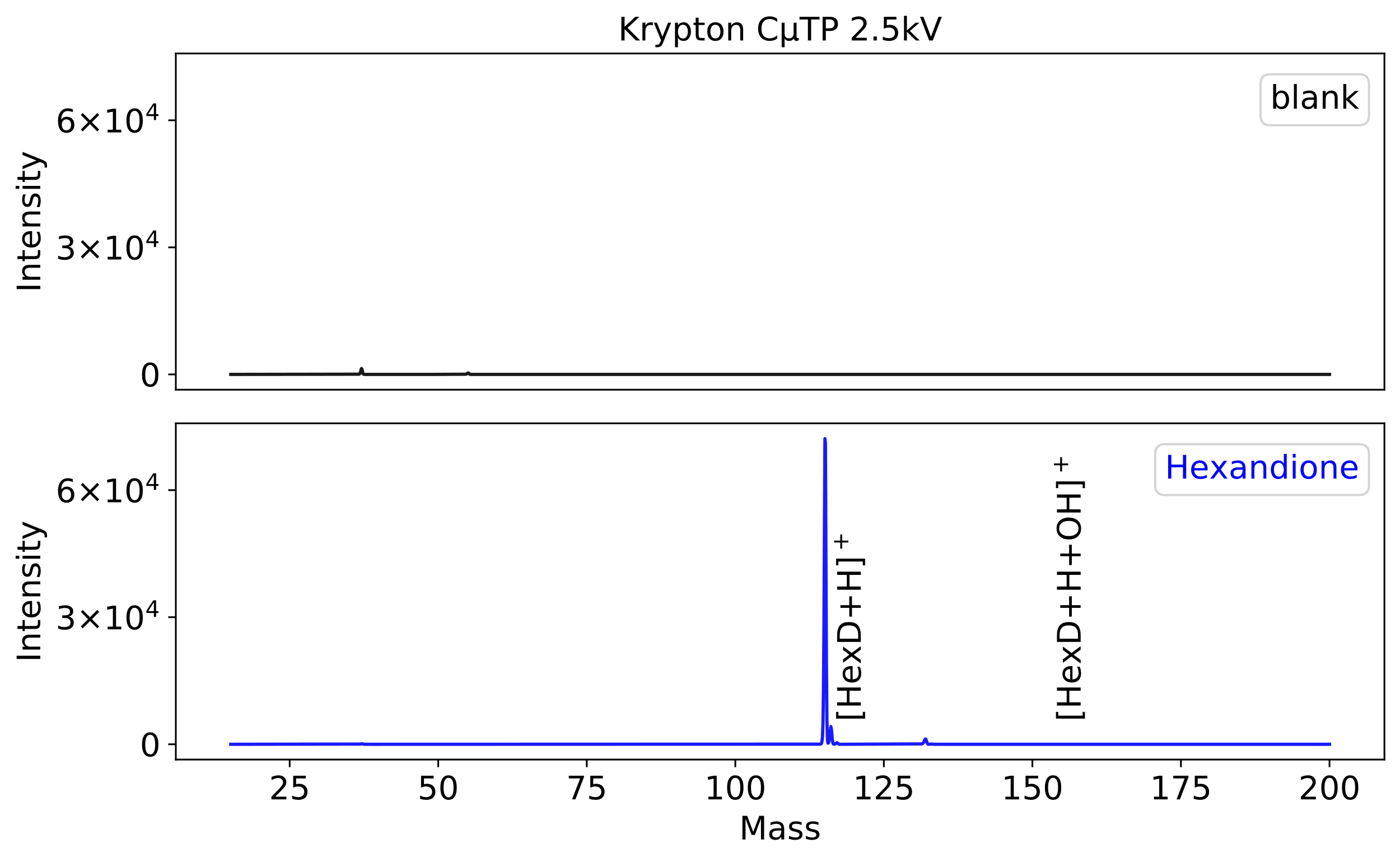

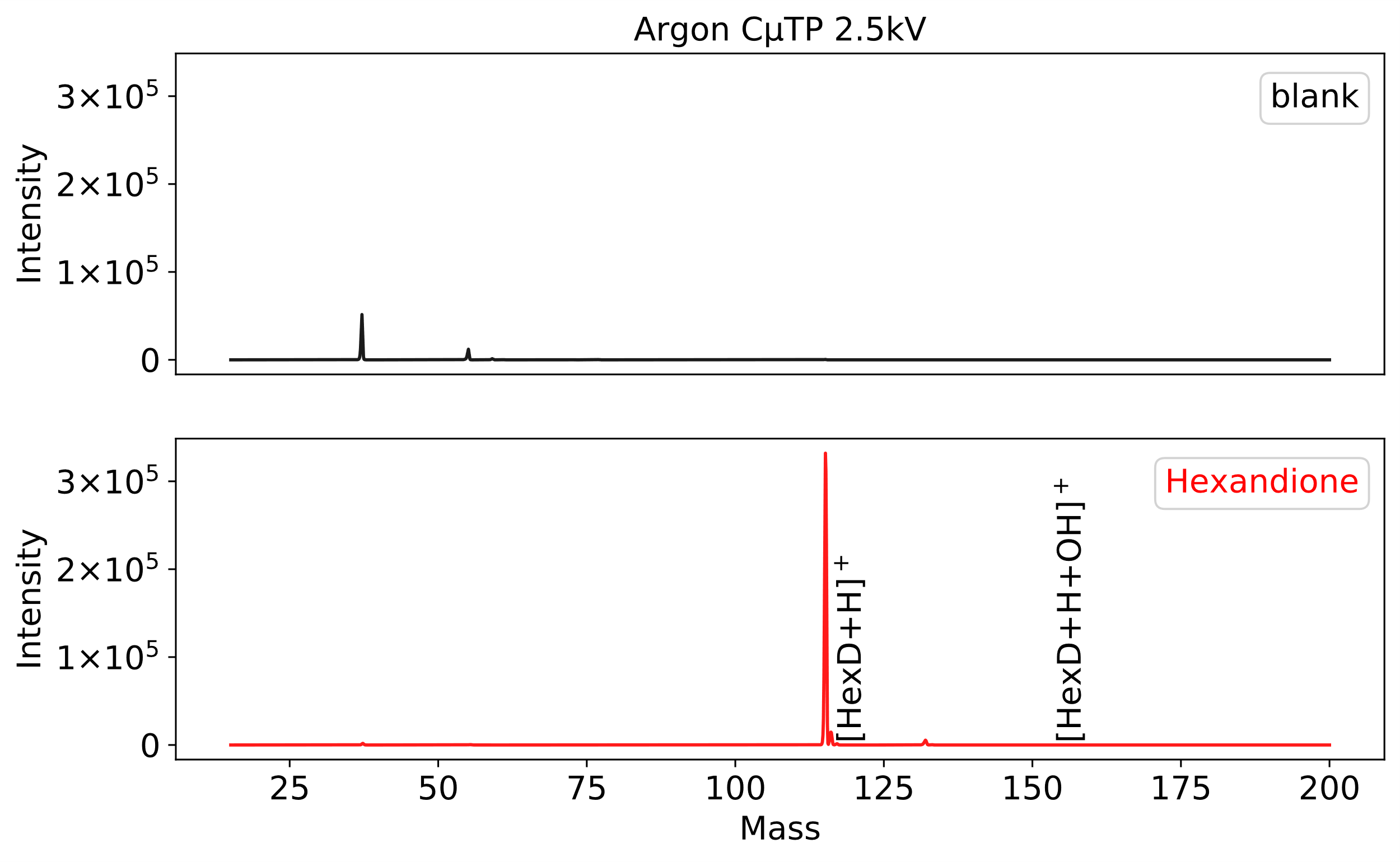

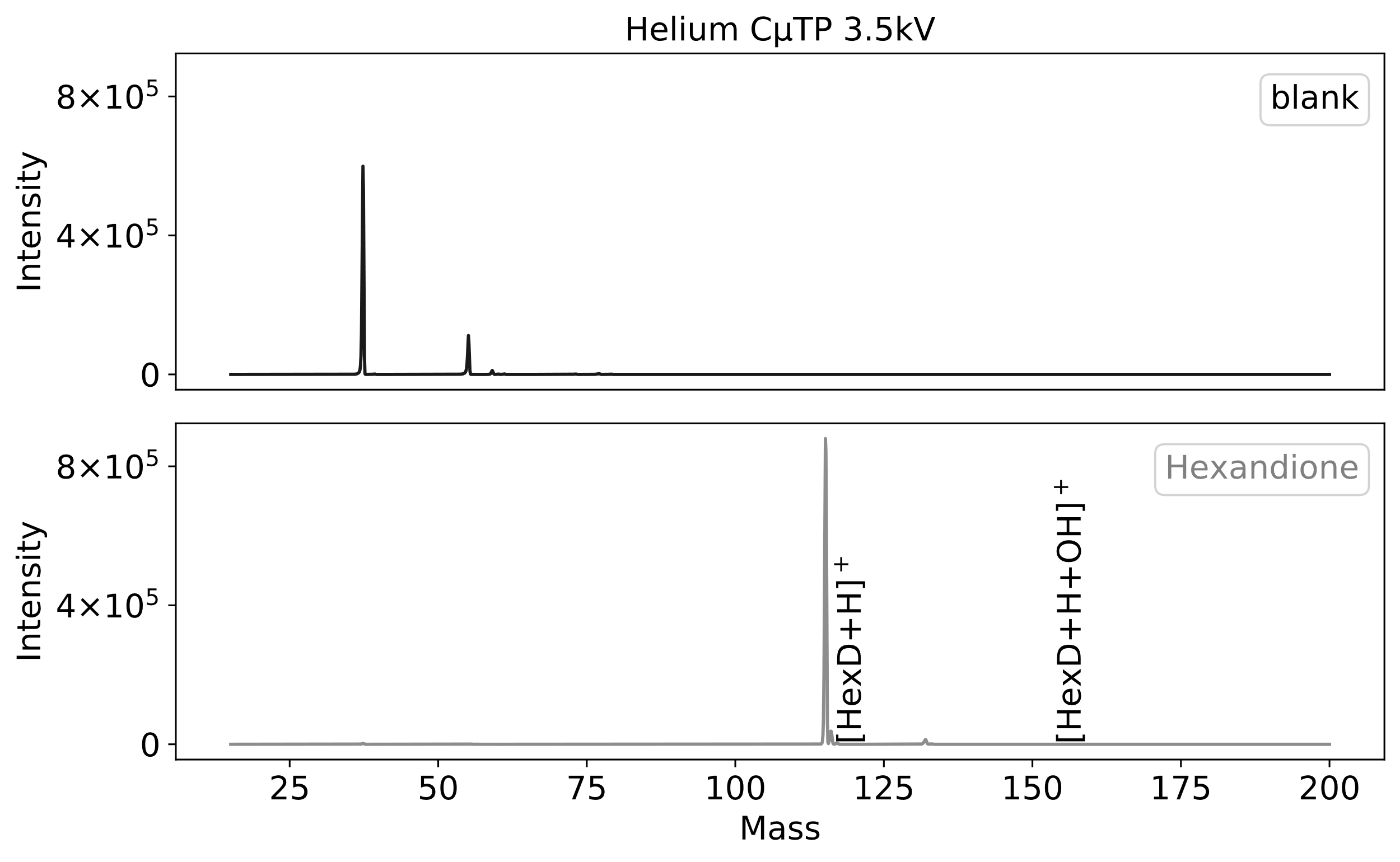

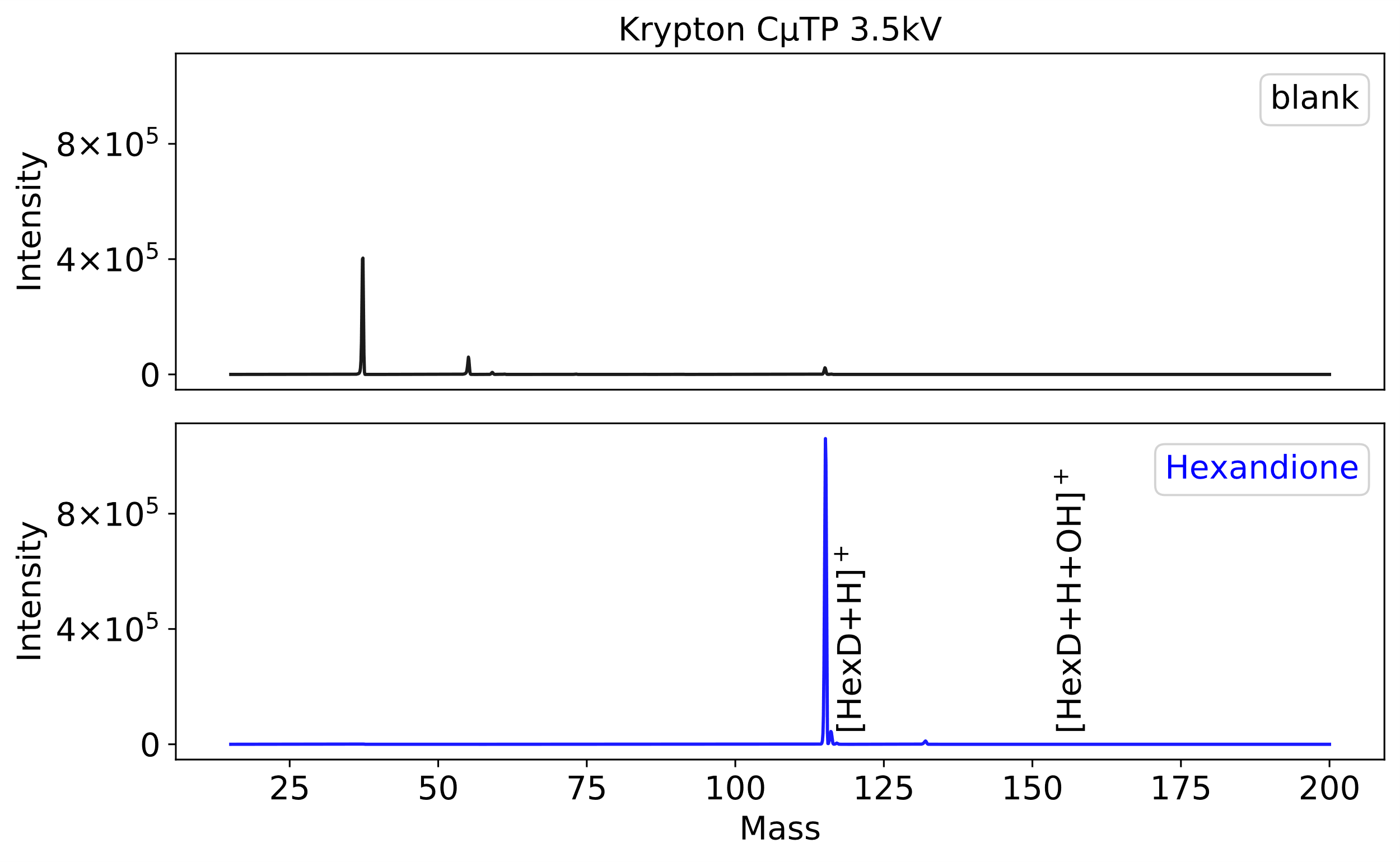

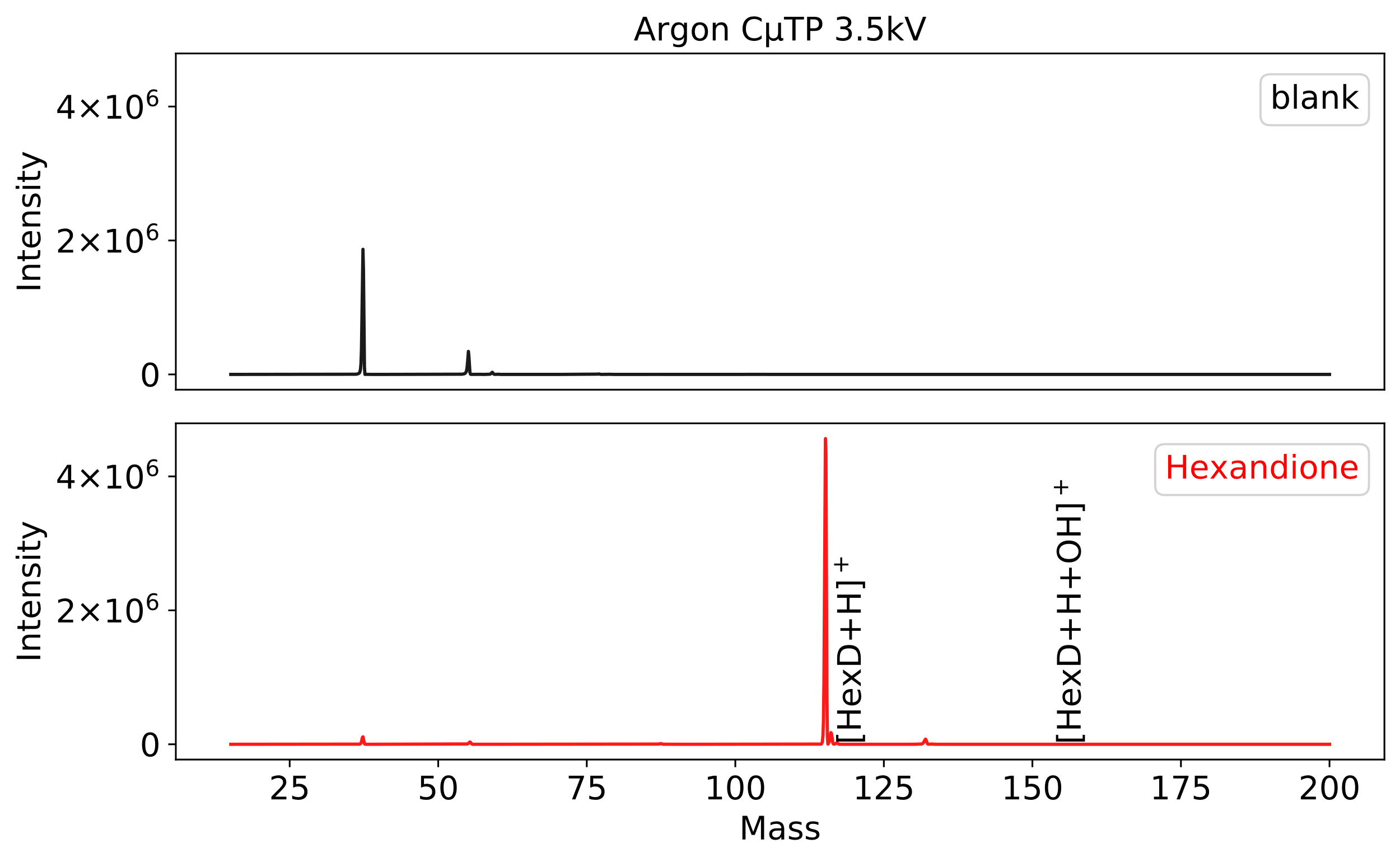


**Figure S4**. Blank- and analyte-spectra of Hexandion when the ionisation sources He-, Ar- and Kr-**CµTP** are operated by 2.5 kV and 3.0 kV.

**Figure S5.** Peak intensities of [H_2_O]_2_+H^+^, [H_2_O]_3_+H^+^, [HexD]+H^+^ and [HexD]+OH+H^+^ obtained with He-, Ne-, Ar- and Kr- **CµTP** sources operated by 2.5 kV and 3.5 kV.


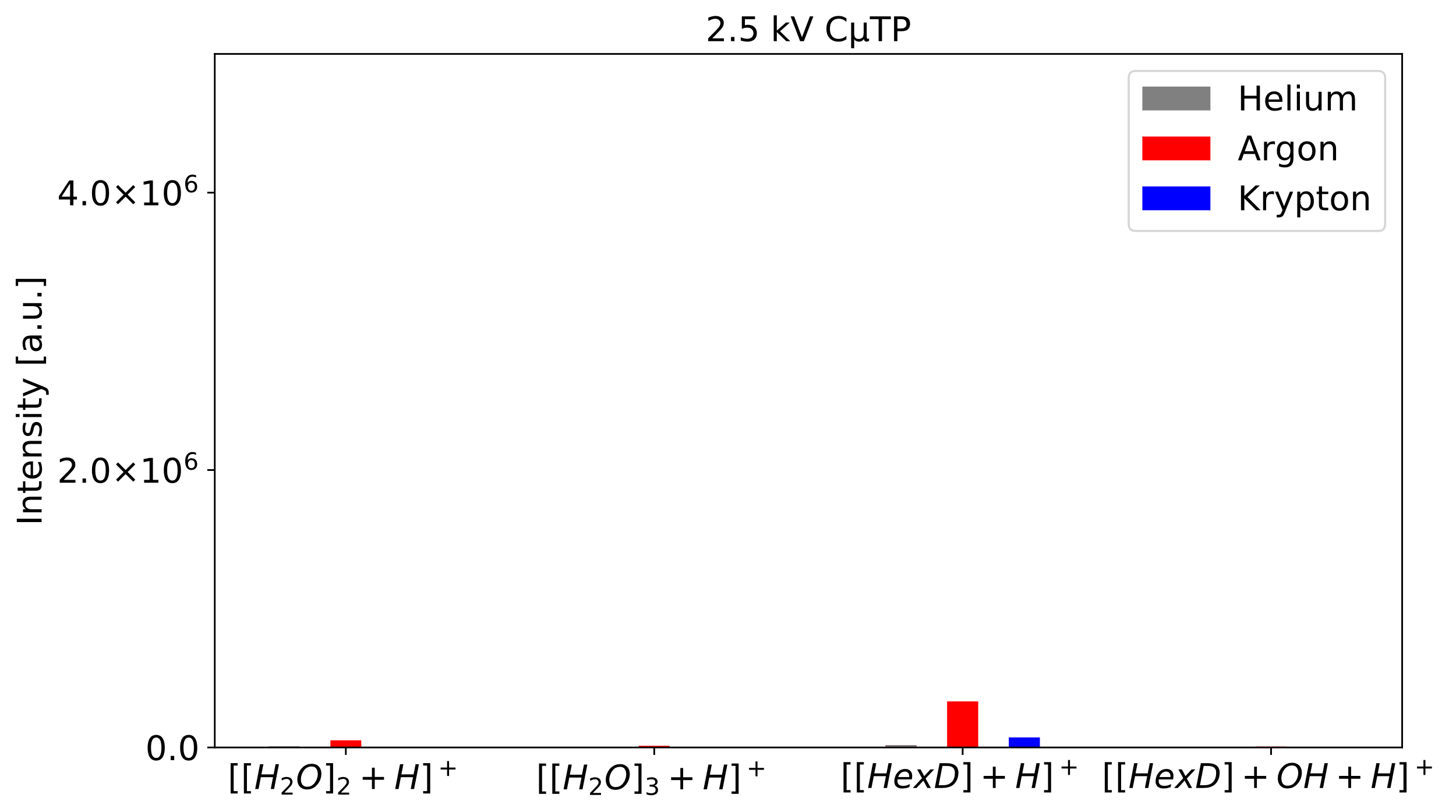

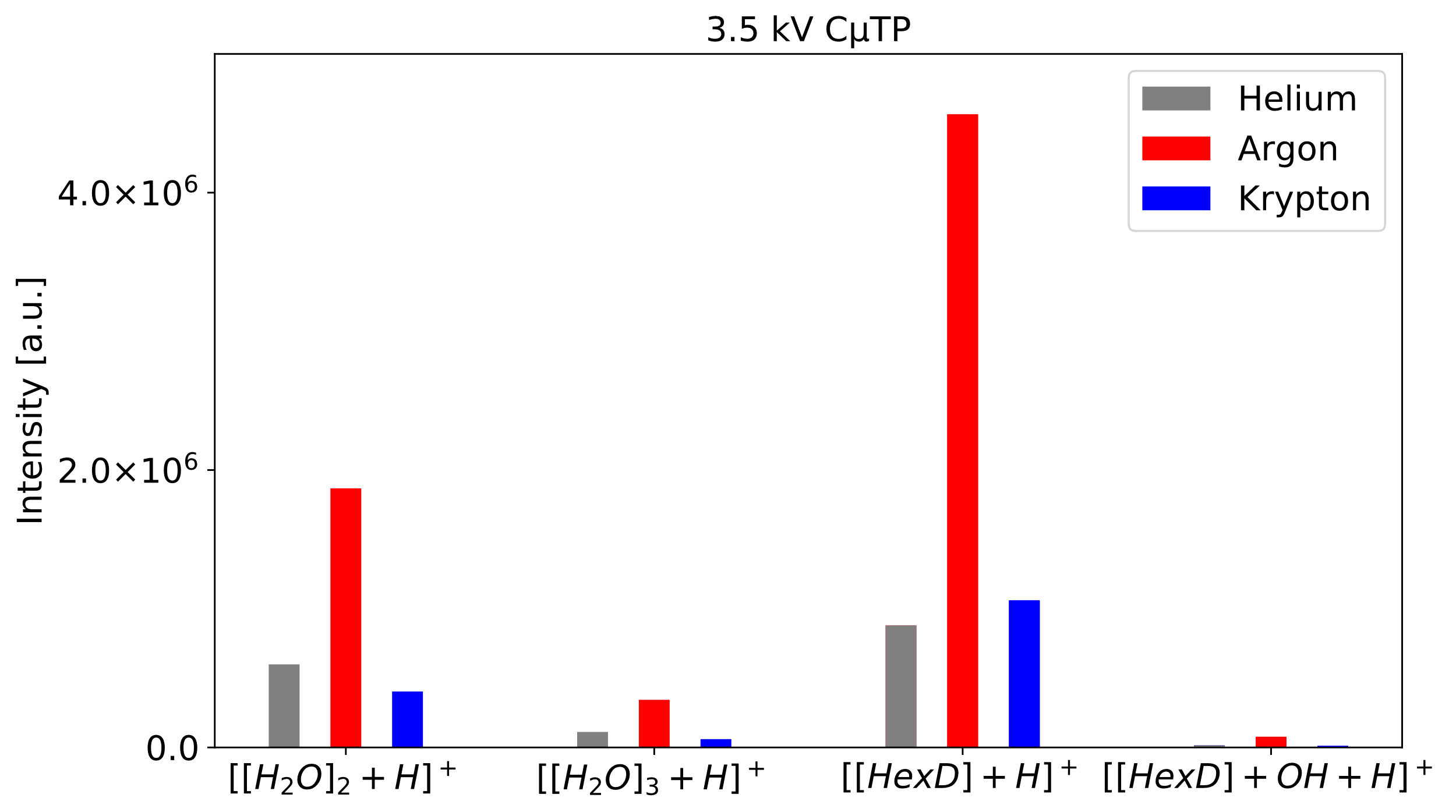

Supplement: Supplementary file 1 — Supplementary file1 (DOCX 5633 KB) [file 216_2024_5420_MOESM1_ESM.docx]
